# Gladiolin produced by pathogenic *Burkholderia* synergizes with amphotericin B through membrane lipid rearrangements

**DOI:** 10.1128/mbio.02611-24

**Published:** 2024-10-18

**Authors:** Claudia Simm, Tzong-Hsien Lee, Harshini Weerasinghe, Dean Walsh, Ioanna T. Nakou, Madhu Shankar, Wai Chung Tse, Yu Zhang, Rebecca Inman, Roger J. Mulder, Freya Harrison, Marie-Isabel Aguilar, Gregory L. Challis, Ana Traven

**Affiliations:** 1 Department of Biochemistry and Molecular Biology and the Infection Program, Biomedicine Discovery Institute, Monash University, Clayton, Victoria, Australia; 2 Centre to Impact AMR, Monash University, Clayton, Victoria, Australia; 3 School of Life Sciences, University of Warwick, Coventry, United Kingdom; 4 Department of Chemistry, University of Warwick, Coventry, United Kingdom; 5 School of Medicine, Monash University, Clayton, Victoria, Australia; 6 Medical Research Council Centre for Medical Mycology, University of Exeter, Exeter, United Kingdom; 7 CSIRO Manufacturing, Research Way, Clayton, Victoria, Australia; 8 ARC Centre of Excellence for Innovations in Peptide and Protein Science, Monash University, Clayton, Victoria, Australia; Geisel School of Medicine at Dartmouth, Hanover, New Hampshire, USA

**Keywords:** *Candida albicans*, amphotericin B, antifungal agents, natural product, Cryptococcus, polyketide

## Abstract

Amphotericin B (AmpB) is an effective but toxic antifungal drug. Thus, improving its activity/toxicity relationship is of interest. AmpB disrupts fungal membranes by two proposed mechanisms: ergosterol sequestration from the membrane and pore formation. Whether these two mechanisms operate in conjunction and how they could be potentiated remains to be fully understood. Here, we report that gladiolin, a polyketide antibiotic produced by *Burkholderia gladioli*, is a strong potentiator of AmpB and acts synergistically against *Cryptococcus* and *Candida* species, including drug-resistant *C. auris*. Gladiolin also synergizes with AmpB against drug-resistant fungal biofilms, while exerting no mammalian cytotoxicity. To explain the mechanism of synergy, we show that gladiolin interacts with membranes via a previously unreported binding mode for polyketides. Moreover, gladiolin modulates lipid binding by AmpB and, in combination, causes faster and more pronounced lipid rearrangements relative to AmpB alone which include membrane thinning consistent with ergosterol extraction, areas of thickening, pore formation, and increased membrane destruction. These biophysical data provide evidence of a functional interaction between gladiolin and AmpB at the membrane interface. The data further indicate that the two proposed AmpB mechanisms (ergosterol sequestration and pore formation) act in conjunction to disrupt membranes, and that gladiolin synergizes by enhancing both mechanisms. Collectively, our findings shed light on AmpB’s mechanism of action and characterize gladiolin as an AmpB potentiator, showing an antifungal mechanism distinct from its proposed antibiotic activity. We shed light on the synergistic mechanism at the membrane, and provide insights into potentiation strategies to improve AmpB’s activity/toxicity relationship.

**IMPORTANCE:**

Amphotericin B (AmpB) is one of the oldest antifungal drugs in clinical use. It is an effective therapeutic, but it comes with toxicity issues due to the similarities between its fungal target (the membrane lipid ergosterol) and its mammalian counterpart (cholesterol). One strategy to improve its activity/toxicity relationship is by combinatorial therapy with potentiators, which would enable a lower therapeutic dose of AmpB. Here, we report on the discovery of the antibiotic gladiolin as a potentiator of AmpB against several priority human fungal pathogens and fungal biofilms, with no increased toxicity against mammalian cells. We show that gladiolin potentiates AmpB by increasing and accelerating membrane damage. Our findings also provide insights into the on-going debate about the mechanism of action of AmpB by indicating that both proposed mechanisms, extraction of ergosterol from membranes and pore formation, are potentiated by gladiolin.

## INTRODUCTION

Fungal diseases are a global health threat with more than a billion infections and a recent estimate by Denning of 2.5 million deaths per year (1). The patient group is no longer restricted to immunocompromised individuals but is extending to chronic conditions such as asthma, chronic obstructive pulmonary disease, cystic fibrosis, diabetes, as well as fungal co-infections in respiratory diseases such as influenza and COVID-19 (2, 3). Treatment options for fungal infections remain scarce, with only three major groups of antifungals (azoles, polyenes, and echinocandins) currently prescribed for invasive infection. Drug-resistant clinical isolates across fungal pathogen species, such as those with acquired mutations in the ergosterol (azoles) and glucan synthase (echinocandins) pathways or, in some cases, with intrinsic physiological resistance, are of growing concern (4–6).

Antifungals of the polyene class were the first to be developed as therapeutics, and the regulatory approval of amphotericin B (AmpB) 63 years ago revolutionized the treatment and survival of patients with invasive fungal infections (7). Despite decades of use as a broad-spectrum antifungal, fungi have acquired little AmpB resistance (7, 8). The reason for this is AmpB’s mode-of-action (MOA), which does not interfere with the activity of an enzyme but rather involves binding to ergosterol in fungal membranes. Traditionally, AmpB’s MOA has been associated with pore formation. AmpB binds ergosterol, causing 4–12 AmpB molecules to aggregate by forming ion channels, which then permeabilize membranes and facilitate the leakage of cellular content (9, 10). However, newer findings have questioned the pore formation mechanism, suggesting that the binding and extraction of ergosterol from the lipid bilayer (i.e., working as a “sterol sponge*”*) is AmpB’s key fungicidal mechanism (11, 12). The sponge model is further supported by the MOA of natamycin, a polyene that is unable to form pores but relies on ergosterol sequestration (13), although recent imaging of AmpB molecules within the fungal membrane is consistent with pore formation (14). Collectively, these studies show that a combination of these two mechanisms is likely important for AmpB’s antifungal activity, but this remains to be fully elucidated.

While AmpB shows broad-spectrum activity and high potency, it displays mammalian toxicity due to the similar structures of fungal ergosterol and its mammalian counterpart cholesterol, as well as other host-derived responses to the drug resulting in nephrotoxicity and additional adverse effects during treatment (7, 15, 16). It is, therefore, challenging to administer AmpB to the critically ill, the very patient group most susceptible to fungal infections (16). Nevertheless, AmpB is the initial treatment choice for *Cryptococcus* infections (4, 17). It is recommended for the treatment of *Candida* infections if echinocandin or azole therapy is not possible, for example, due to resistance or lack of commercial availability (18). It is also prescribed for azole-resistant *Aspergillus* infections (19), as well as other rarer and difficult to treat infections caused by fungi including *Histoplasma* and *Mucor* (7). Moreover, AmpB is becoming increasingly important for treating infections caused by *Candida auris* due to the worrying emergence of strains resistant to both azoles and echinocandins (20). Lipid formulations of AmpB reduce toxicity, and recent efforts have leveraged insights from the sterol sponge model to introduce structural changes in the AmpB molecule that resulted in a less toxic compound with promise against fungal infections in pre-clinical mouse infection studies (15). Notwithstanding these recent advances, additional strategies are needed to improve the activity/toxicity relationship for AmpB (7), and the 2022 WHO Fungal Priority Pathogens report called not only for new drug development but also for insights into potentiation strategies that could be used in combination with current drugs to improve health outcomes (21). Various attempts to identify suitable potentiators of AmpB have been made, with reduced AmpB aggregation being described for some potentiators and the mechanism of potentiation remaining largely unknown for others (22–26).

Here, we report that gladiolin, a polyketide antibiotic produced by the opportunistic bacterial pathogen *Burkholderia gladioli* (27), is a potentiator of AmpB against WHO critical or high priority fungal pathogens *C. albicans*, *C. neoformans*, *C. auris,* and *C. glabrata*. By studying gladiolin’s antifungal activity, we show a distinct MOA from that associated with its antibiotic activity and present a membrane-based mechanism of AmpB potentiation. Our findings provide evidence for both pore formation and structural membrane rearrangements by the synergistic combination of AmpB and gladiolin, which together result in accelerated and increased membrane damage.

## RESULTS

### Gladiolin synergizes with AmpB against several priority fungal pathogens

**Fig 1:**
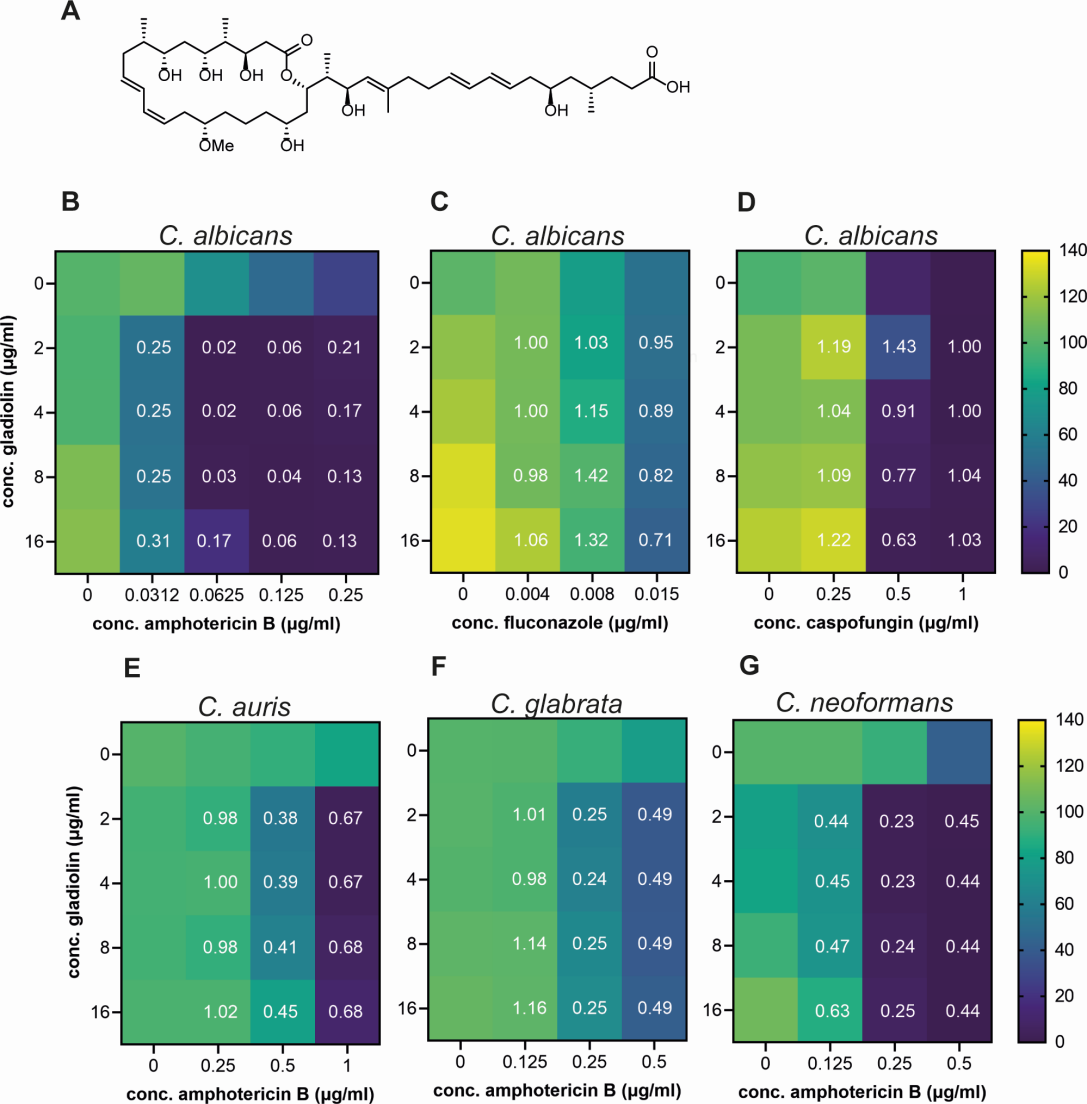
Gladiolin synergizes with AmpB against multiple priority fungal pathogens. (**A**) Chemical structure of gladiolin. Heatmaps of checkerboard assays of gladiolin in combination with (**B**) amphotericin, (**C**) fluconazole, and (**D**) caspofungin. Shown here are the data for the reference strain SC5314, while an additional 20 clinical isolates are shown in Fig. S2A. (**E**) Heatmap of gladiolin/AmpB checkerboard assays for *C. auris* 470140 (clade I). Additional *C. auris* clades are shown in Fig. S2B. (**F**) Heatmap of gladiolin/AmpB checkerboard assay for *C. glabrata* ATCC2001. (**G**) Heatmap of gladiolin/AmpB checkerboard assays for *C. neoformans* H99. For all checkerboard experiments, fungal cultures were grown in RPMI, pH 7.0, and cell density at 600 nm was measured at 20 h of growth at 37°C for all *Candida* species and at 48 h for *Cryptococcus*. The color scale shows the percentage of survival compared to untreated control. The fractional inhibitory concentration index (FICI) is indicated as white numbers and is defined as <0.5 (synergistic), >0.5 to <1 (additive), 1–4 (indifferent), and >4 (antagonistic). Heatmaps and FICIs were derived from mean values of 3–4 biological repeats.

The gladiolin-producing *Burkholderia gladioli* strain was isolated from a cystic fibrosis (CF) lung (27), where *Candida albicans* can also be found (28). The absence of *Candida* spp. in CF patients infected with *Burkholderia* species (29, 30) prompted us to perform a detailed study of gladiolin’s potential antifungal activity. Minimum inhibitory concentration (MIC) assays of gladiolin (structure shown in Fig. 1A) were performed using the *C. albicans* reference strain SC5314 as well as a panel of other clinical isolates (31). Fungal cultures were grown in RPMI with 10 mM glucose in the presence of increasing concentrations of gladiolin either under hyphal (RPMI, pH 7.0, 37°C) or yeast growth conditions (RPMI, pH 5.6, 30°C). On its own, gladiolin did not inhibit the growth of any of the 21 *C*. *albicans* strains that we tested (Fig. S1A and D) in contrast to a previous report (27). No effect on growth was observed when non-glucose carbon sources were used (galactose or mannose) or when we reduced the glucose concentration to mimic the low glucose environment of the lung (Fig. S1E). Increased optical density (OD) of gladiolin-treated cultures in hyphae-inducing conditions was seen for a number of clinical *C. albicans* isolates that form substantial hyphae (such as SC5314, P87, and P76067) (Fig. S1A and D) and was due to gladiolin’s effect on *C. albicans* cell morphology. Gladiolin-treated cultures were a homogenous, turbid cell suspension consistent with yeast morphology, while control cultures appeared less turbid and showed hyphal aggregations (Fig. S1B). Microscopy confirmed that gladiolin-treated cultures exhibited a larger proportion of yeast cells relative to the hyphal controls (Fig. S1C).

Next, we investigated the activity of gladiolin in combination with representatives of the three major classes of antifungals: the azole fluconazole (inhibits ergosterol biosynthesis), the echinocandin caspofungin (inhibits glucan synthase), and the ergosterol-binding polyene AmpB (causes membrane disruption). Checkerboard experiments of compound combinations were performed, and the fractional inhibitory concentration index (FICI) was calculated (32, 33). These experiments revealed that gladiolin displays strong synergism with AmpB with FICIs of 0.02–0.42 against all *C. albicans* clinical isolates tested (Fig. 1B; Fig. S2A; Table S1). In contrast, gladiolin did not synergize with fluconazole (Fig. 1C) or caspofungin (Fig. 1D). Gladiolin also strongly synergized with AmpB against *C. neoformans*, *C. auris,* and *C. glabrata* (Fig. 1E through G) and against strains across clades I–IV of *C. auris* including an echinocandin-resistant isolate from clade I (Fig. S2B).

**Fig 2:**
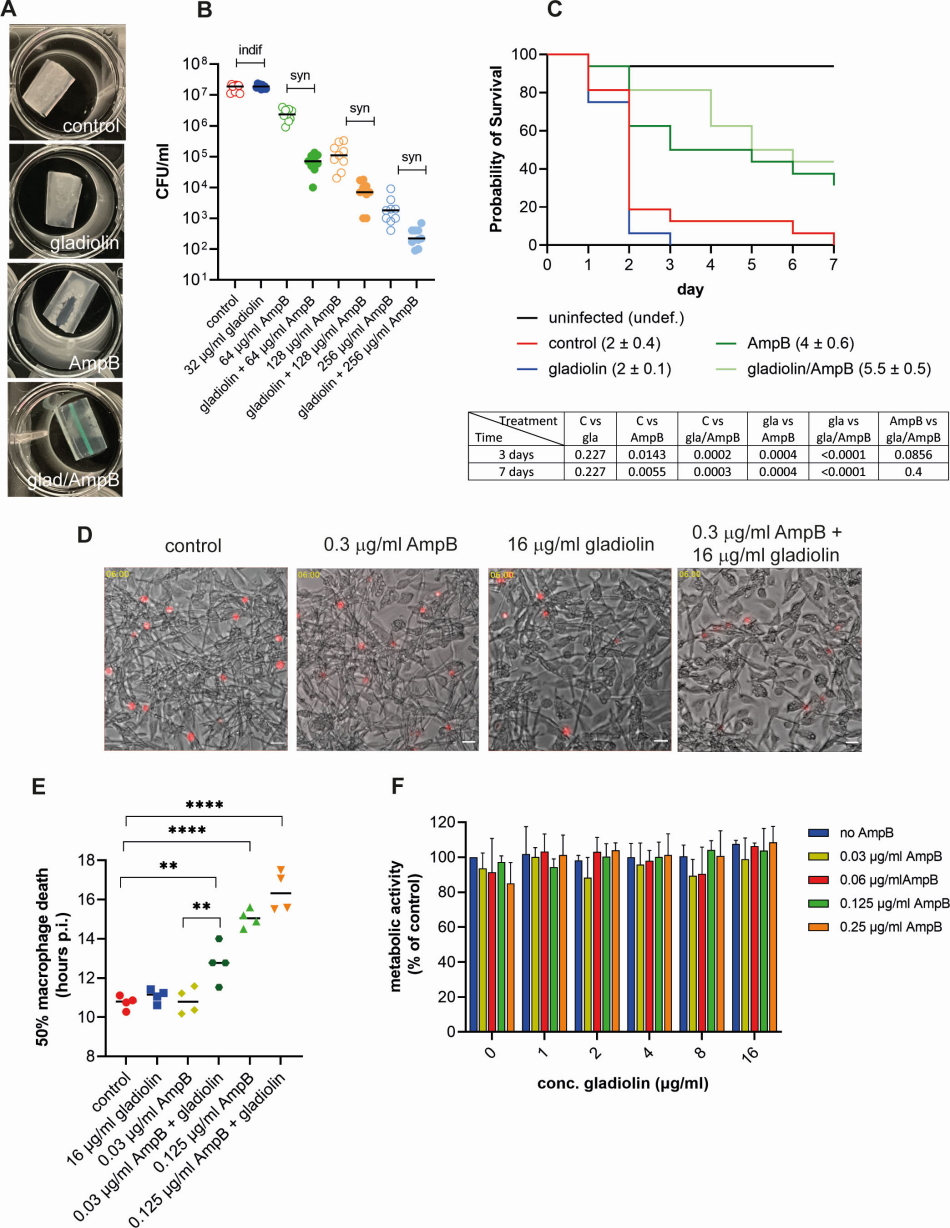
Combinatorial effects of gladiolin with AmpB against drug-resistant fungal biofilms, and during immune cell and animal infections. (**A**) *C. albicans* biofilms were grown in synthetic ventilator airway mucus (SVAM) medium on endotracheal tubes cuttings for 48 h (37°C and 5% CO_2_). Biofilms were treated for 24 h as indicated before being photographed. (**B**) Experiment as in panel A. Biofilms were dislodged after drug treatment and plated for CFUs. Data were generated from three biological repeats with three technical repeats each. Synergistic interactions were evaluated by using the Response-Additivity model as described in the Materials and Methods (“indif” is indifferent and “syn” is synergistic). (**C**) Larvae of *Galleria mellonella* were infected with *C. albicans* and treated with 50 µg/mL AmpB, 32 µg/mL gladiolin, or the combination of both. Larvae were monitored for survival over 7 days. Data were derived from four independent experiments with four larvae per condition and statistically analyzed using the log-rank Mantel-Cox test with *P*-values at days 3 and 7 shown underneath. The numbers in parenthesis represent the median survival in days. (**D**) The filamentation of *C. albicans* during the challenge of mouse bone marrow-derived macrophages (BMDMs) at 6 h post infection. In control conditions, abundant escaped hyphae are visible in the medium surrounding macrophages. There is a small reduction in visible escaped hyphae in the presence of gladiolin and an additional reduction in the gladiolin/AmpB combination. Images are stills taken from live cell imaging movies used to generate data in panel E. Shown is the overlay of bright field and Draq7-stained nuclei of macrophages that were lysed by the growing fungal hyphae. Images were cropped and adjusted for brightness. The scale bar shows 100 µm. (**E**) Macrophage death of *C. albicans*-infected BMDMs (MOI 3:1) with combinations of gladiolin and AmpB treatment was determined by Draq7 staining. A minimum of 900 macrophages were counted per experiment for each of the conditions. The half-maximal macrophage death of two independent experiments with two technical repeats each was determined using 4-parameter analysis and statistically evaluated using a one-way ANOVA with Dunnett’s multiple comparison test (**P < 0.01, ****P < 0.0001). (**F**) Metabolic activity of uninfected BMDMs treated for 24 h with single or drug combinations was measured using resazurin metabolic assay, and the error bars indicate SEM with *n* = 3.

Since the gladiolin-producing *Burkholderia gladioli* was isolated from a CF lung infection (patient sputum culture), we investigated the activity of gladiolin under conditions mimicking this environment. *C. albicans* does not cause pneumonia, but it is found in the respiratory tract (34), and some evidence suggests that *C. albicans* may impact the severity of diseases such as ventilator-associated bacterial pneumonia, possibly through fungal-bacterial interactions that modulate bacterial virulence (34, 35). We, therefore, tested the gladiolin/AmpB drug combination in our recently developed model of ventilator-associated pneumonia (36), which uses complex synthetic ventilator airway mucus (SVAM) medium to mimic host conditions. Gladiolin also synergized with AmpB when applied to established drug-resistant fungal biofilms in this model (Fig. 2A and B). This shows that gladiolin potentiates AmpB against a structurally complex, drug-resistant fungal biofilm grown in a nutritional environment expected to be encountered in host tissues. Similarly, gladiolin showed a trend toward potentiation of AmpB in the *Galleria* infection model (Fig. 2C). The median larvae survival was extended from 4 days for AmpB alone to 5.5 days for AmpB in combination with gladiolin.

While *C. albicans* does not utilize macrophages as a replication niche, it escapes from them and kills them by several mechanisms, reviewed in reference (37). In combination with AmpB, gladiolin slowed the escape of *C. albicans* hyphae from macrophages (Fig. 2D), and the addition of just 0.03 µg/mL AmpB together with 16 µg/mL gladiolin delayed the time to reach 50% macrophage cell death by 2 h (Fig. 2E), showing that the combination slows fungal growth. This delay is entirely attributed to the compounds acting in combination since neither gladiolin nor 0.03 µg/mL AmpB on their own showed any difference in the timing of macrophage cell death compared to the negative control. Increasing the AmpB concentration to 0.125 µg/mL extended the delay in macrophage cell death to 4.5 h, but this was not further enhanced by gladiolin (Fig. 2E). Neither gladiolin nor AmpB alone or in combination caused a reduction of metabolic activity in primary mouse bone marrow-derived macrophages (BMDMs) (Fig. 2F). This result shows that, despite synergizing with AmpB against fungi, gladiolin does not exacerbate the cytotoxic effects of AmpB on mammalian cells.

### Potentiation of AmpB by gladiolin relies on membrane permeabilization but not on direct physical association in solution

**Fig 3:**
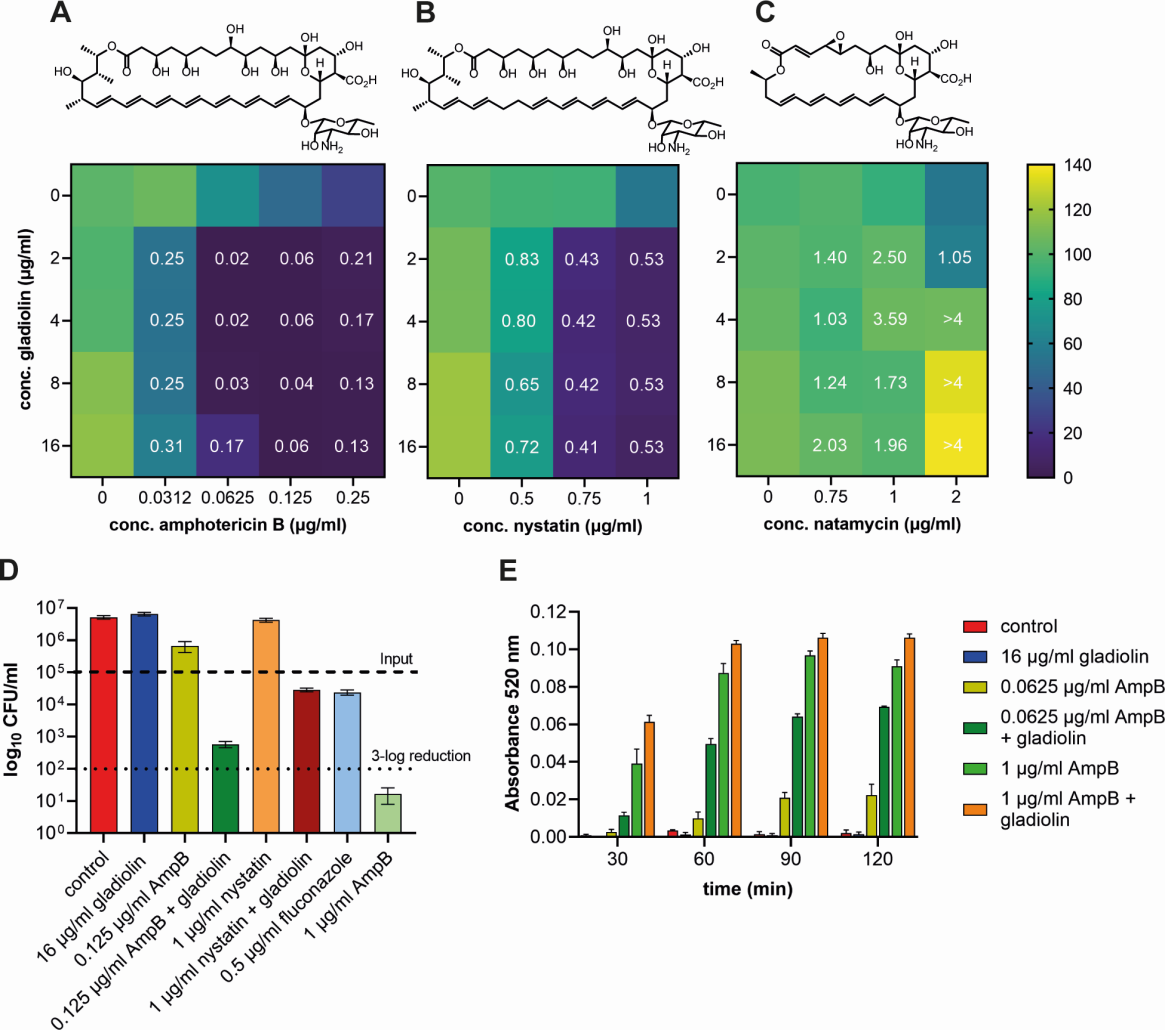
Gladiolin acts synergistically with AmpB and nystatin but not with natamycin. Heatmaps of checkerboard assays of gladiolin in combination with (**A**) AmpB, (**B**) nystatin, and (**C**) natamycin. *C. albicans* cultures were grown in RPMI, pH 7.0, and OD_600_ was measured after 20 h of growth at 37°C. The color scale shows the percentage of survival compared to the untreated control. The fractional inhibitory concentration index (FICI) is indicated as white numbers and is defined as <0.5 (synergistic), >0.5 to <1 (additive), 1–4 (indifferent), and >4 (antagonistic). Heatmaps and FICIs were derived from mean values of four biological repeats. The heatmap for AmpB in A is the same as in Fig. 1 and is shown here again for direct comparison with the other two polyenes. (**D**) *C. albicans* cultures were grown as described above. After 20 h of incubation, aliquots were diluted appropriately and plated onto YPD plates. CFUs were counted after 2 days of incubation at 30°C. Error bars indicate SEM (*n* = 3), and the dashed lines indicate the initial inoculum and its 3-log reduction, respectively. (**E**) Efflux of rhodamine 6G (R6G). *C. albicans* cells were loaded with R6G for 1 h and subsequently resuspended in dye-free PBS. The efflux was measured by the increase of absorbance at 520 nm over time. The error bars indicate SEM (*n* = 3).

The classical model of AmpB’s MOA involves ergosterol binding and pore formation (14). A similar MOA has been predicted for nystatin, but the much shorter polyene natamycin is unable to form ion channels (7, 13). Therefore, to obtain a better understanding of the synergistic MOA of gladiolin and AmpB, we tested if nystatin and natamycin could also interact synergistically with gladiolin. While nystatin showed synergistic effects at 0.75 µg/mL and additive effects at 0.5 and 1 µg/mL, no potentiation could be observed for natamycin at all concentrations tested (Fig. 3A through C). These results suggest that pore formation by AmpB and nystatin is involved in the synergistic MOA. Next, we tested if a reduction in AmpB concentration in combination with gladiolin would result in fungicidal killing. Cultures treated with 0.125 µg/mL AmpB, a concentration that causes a 10% reduction in colony-forming units (CFUs) compared to control, were further inhibited by the addition of gladiolin to near 3-log reduction (99.5%) of the initial inoculum (Fig. 3D). AmpB at 1 µg/mL proved to be fungicidal with a 3-log reduction (99.9%), while gladiolin on its own did not show inhibition in CFUs confirming previous MIC experiments. The subtoxic effect of 1 µg/mL nystatin could be enhanced by gladiolin to the same level as the fungistatic drug fluconazole at its MIC (Fig. 3D).

To investigate whether direct interaction between gladiolin and AmpB is responsible for the observed synergism, we used NMR diffusion-ordered spectroscopy (DOSY) (Fig. S3A and B). These and all subsequent biophysical experiments were performed with AmpB-deoxycholate (fungizone) due to its higher solubility which allowed us to reduce the volume of DMSO used as the solvent, resulting in a less intense protiated signal. The log values of the measured diffusion coefficients for AmpB and gladiolin are −9.48 and −9.69, respectively. The same values were measured for a mixture of the two compounds, indicating that they are well-separated entities that do not physically associate in solution (see the supplemental material, ^1^H and DOSY NMR results). We also compared the ability of AmpB and AmpB-deoxycholate (fungizone) to synergize with gladiolin and found no difference between the two formulations (Fig. S3C). Moreover, deoxycholate at 12.5 µg/mL did not synergize with gladiolin even though slight growth inhibition was observed at this concentration (Fig. S3D). Collectively, these results indicate that gladiolin does not act simply as a detergent to solubilize AmpB to cause enhanced membrane interactions and antifungal efficacy.

### Gladiolin interacts with model membranes and modulates membrane interactions of AmpB

**Fig 4:**
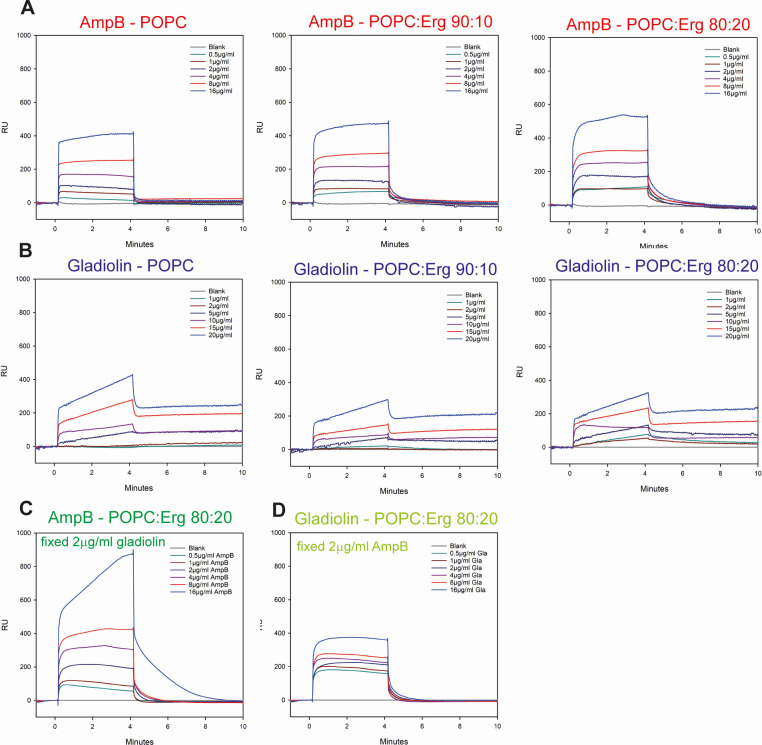
Gladiolin interacts with model membranes and modulates membrane binding by AmpB. Dose-response analysis of (**A**) AmpB or (**B**) gladiolin binding to model lipids. Sensorgrams were obtained from POPC or POPC:ergosterol model lipids with ratios of either 90:10 or 80:20. The drugs were injected in twofold dilution series ranging from 0 to 16 µg/mL. (**C**) SPR using concentrations of AmpB from 0.5 to 16 µg/mL with a fixed concentration of 2 µg/mL gladiolin. (**D**) Same as panel C, but here, gladiolin concentrations varied from 0.5 to 16 µg/mL with a fixed concentration of 2 µg/mL AmpB. The sensorgrams shown are a representative example of three independent replicates.

Next, we took AmpB’s MOA into account and considered the possibility that synergism could be due to gladiolin interacting with lipids, thereby potentiating the ability of AmpB to disrupt membrane integrity. As a first approach to test this hypothesis, we studied efflux rates of *C. albicans* cultures treated with gladiolin, AmpB, or their combination by measuring the release of the dye rhodamine 6G (R6G) from yeast cells (Fig. 3E). The efflux of R6G was enhanced by the addition of gladiolin, in particular for the subtoxic concentration of AmpB (0.0625 µg/mL) over the entire time course, suggesting a combinatorial effect of gladiolin on membrane integrity. To further investigate this, we studied the binding of gladiolin to phosphatidylcholine (POPC) lipid membranes with different quantities of ergosterol (0%–20%) using surface plasmon resonance (SPR) (Fig. 4). Gladiolin and AmpB were applied to model lipid membranes either alone or as a mixture. In accordance with previous findings (38–40), AmpB showed high affinity for model lipid membranes displaying a dose-dependent increase in response units (RUs), and fast association and dissociation rates (Fig. 4A). Elevating ergosterol content increased the amount of binding of AmpB compared to ergosterol-free, 100% POPC membranes. The role of ergosterol in enhanced AmpB binding was further confirmed by slower dissociation rates for AmpB from POPC membranes containing 10% and 20% ergosterol in comparison to POPC alone. The binding of AmpB to the lipid membranes did not reach saturation in the concentration range tested (0.5–16 µg/mL).

Gladiolin also interacted with the model membranes (Fig. 4B). However, its binding kinetics are biphasic, showing rapid initial association (potentially an interaction phase) followed by a slower association (potentially an insertion phase). Gladiolin’s binding to model lipids occurs in a dose-dependent manner. However, unlike AmpB, no enhanced gladiolin binding was observed for the ergosterol-POPC membranes. Instead, the highest RUs were seen for POPC membranes without ergosterol. The dissociation of gladiolin from model lipids was biphasic and partially irreversible.

In combination experiments, AmpB binding was enhanced by the addition of 2 µg/mL gladiolin as indicated by higher RUs (Fig. 4C). For AmpB concentrations between 0.5 and 8 µg/mL, the binding kinetics in the presence of gladiolin were similar to those of AmpB on its own (fast association followed by fast dissociation). The highest concentration of AmpB tested (16 µg/mL) in combination with gladiolin showed a biphasic SPR profile similar to that of gladiolin alone with a fast initial and a slower subsequent association phase. In addition, the dissociation was slower than observed for AmpB on its own. We also performed the opposite experiment, combining a constant concentration of AmpB (2 µg/mL) with increasing concentrations of gladiolin (Fig. 4D). In this case, the previously observed biphasic SPR profile for gladiolin was no longer detectable. Instead, the association/dissociation curve was similar to that of AmpB alone and may suggest that the action of AmpB overpowers that of gladiolin in this scenario. Collectively, these data show that gladiolin interacts with model membranes, enhances the ability of AmpB to associate with membranes, and changes the AmpB binding profile.

### Gladiolin accelerates membrane damage caused by AmpB

**Fig 5:**
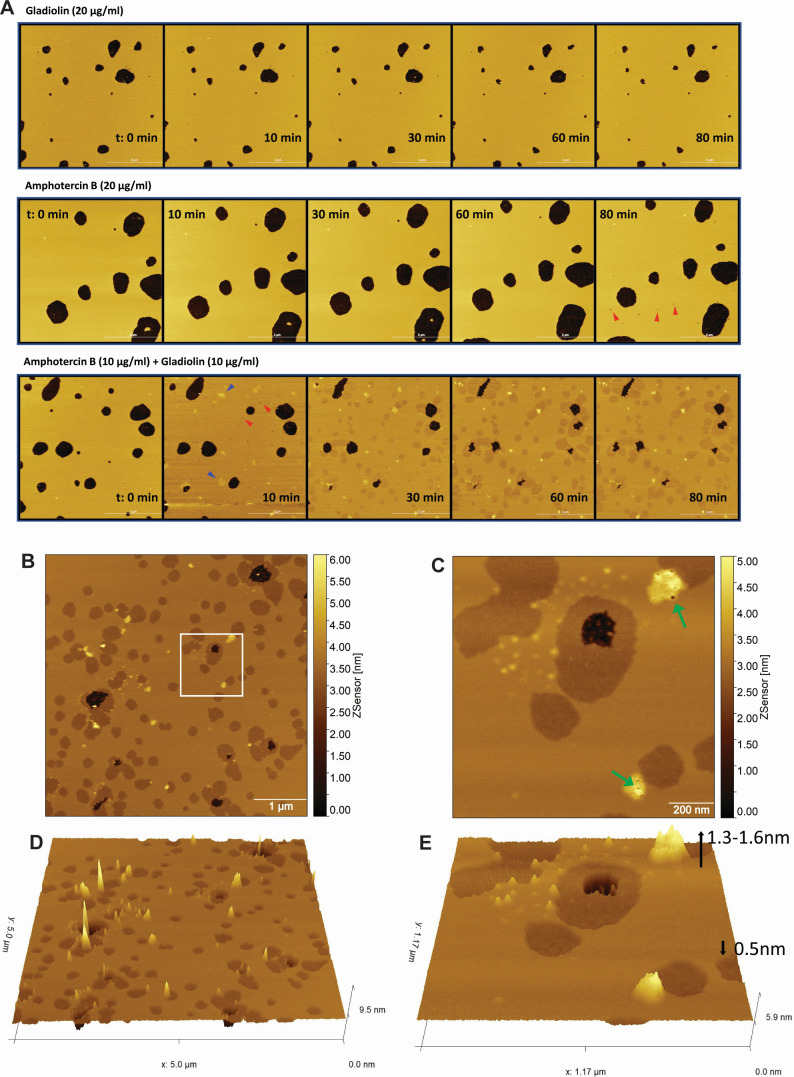
Gladiolin accelerates and exacerbates membrane damage by AmpB. Images show liposome-deposited mica disks composed of POPC:ergosterol (80:20). Black spots indicate areas where supported lipid bilayers (SLBs) have not been formed. (**A**) Time course of the topographical changes of SLBs treated with gladiolin, AmpB, or their combination. SLBs were observed over a time course of 120 min in the presence of 20 µg/mL single drug or a 10 µg/mL each gladiolin/AmpB combination. Images are stills taken from the supplemental movies. Pits with a decreased membrane depth are labeled with red arrows and thickened sections with blue arrows. (**B**) Topographic structure of SLBs with the addition of AmpB (10 µg/mL) and gladiolin (10 µg/mL) mixture imaged at 58–62 min post-addition. (**C**) Zoomed-in topographical profile of white inset box in panel B. The color scale shows the depth of the SLBs with pits appearing in darker colors and areas with increased depth shown in yellow. Green arrows indicate holes of various diameter and depth inside raised structures. (**D and E**) 3D presentation of images B and C, respectively, showing pitted area of lower (in brown) and raised structures with increased height (in yellow).

The effects of AmpB and gladiolin on the structure of membranes were assessed by examining topographic changes of POPC:ergosterol (80:20) supported lipid bilayers (SLBs) using atomic force microscopy (AFM) in a peak force quantitative nanomechanical mode. POPC-ergosterol formed a homogenous layer on mica disks with a thickness of 4.2 nm. The effects of various treatments on SLBs were observed for 120 min, and the SLBs remained stable for the entire time period using DMSO as a negative control condition (Movie S1). No change in the structure of the SLBs was observed during a 120 min period in the presence of 20 µg/mL gladiolin (Fig. 5A, top panel; Movie S2). Upon incubation of the SLBs with 20 µg/mL AmpB, no change in the bilayer structure was observed for the first 60 min (Fig. 5A, middle panel; Movie S3). After 60 min, AmpB induced several small pits with 0.5–0.6 nm indents into the SLBs (marked with red arrows). These pits are consistent with the thinning of the membrane, which has previously been reported to result from ergosterol extraction by AmpB (41). The size and number of these pits in the SLBs continued to increase over time. Several areas of the bilayer with an increased thickness of 1–1.6 nm appeared toward the end of the time course revealing holes with diameters of 5–20 nm in these thickened regions (Movie S3). No holes were observed in the pitted bilayers.

The formation of pitted structures in POPC:ergosterol 80:20 SLBs was accelerated and more drastic when AmpB was combined with gladiolin (Fig. 5A, bottom panel; Movie S4). Pits (marked with red arrows) appeared within 8–10 min of adding the compound combination and were greater than 10 nm in diameter and 0.5–0.6 nm in depth. The size and number of bilayer pits increased over time and gradually merged into large patches. In contrast to the small size of bilayer pits observed for the AmpB alone, the size of the bilayer pits induced by the AmpB/gladiolin mixture was larger and merged into large patches at a much faster rate. Several areas of bilayer with increased thickness of up to 1.3 nm (marked with blue arrows) were observed at the same time as the formation of pits (Fig. 5B through E). These areas of increased thickness showed irregular surfaces with holes of 5–20 nm in diameter and depths of up to 4 nm, indicating destruction of the POPC:ergosterol bilayer (Fig. S4A).

To examine the specificity of the gladiolin/AmpB interactions in membranes, we similarly investigated natamycin, the shorter polyene that does not synergize with gladiolin (Fig. 3C). Natamycin was unable to induce structural changes on its own or in combination with gladiolin (Fig. S4B; Movies S5 and S6) supporting the lack of synergism. Collectively, the AFM imaging shows that the synergistic effect of gladiolin stems from its ability to accelerate and enhance membrane destruction caused by AmpB.

## DISCUSSION

Our study characterizes the antifungal activity of gladiolin, a recently discovered natural product that has promising activity against *Mycobacterium tuberculosis* (27). We report that gladiolin synergizes with AmpB against several important human pathogens (*C. albicans*, *C. neoformans*, *C. auris,* and *C. glabrata*), including against *C. albicans* biofilms and in host infection models. Synergistic antifungal activity of natural products with AmpB has recently been reported; however, their underlying MOAs remain to be directly characterized (42, 43). Interestingly, like gladiolin, these other natural products synergize with AmpB but not with azoles or echinocandins. Our MOA studies using biophysical methods revealed that the synergism of gladiolin with AmpB results from the ability of gladiolin to associate with lipid bilayers and a functional interaction between the two compounds in the membrane. This MOA is distinct from the mechanism associated with the antibacterial activity of gladiolin and structurally related compounds, which results from inhibition of RNA polymerase (27, 44).

We show that gladiolin interacts with model membranes on its own, with biphasic kinetics and in a partially irreversible manner. This is reminiscent of some antimicrobial peptides and toxins (45–48) but, to the best of our knowledge, such an interaction mode has never been reported for a polyketide. Our interpretation of the biphasic binding observed for gladiolin is that it undergoes fast and reversible initial association with the outer leaflet of the membrane and then slowly and irreversibly inserts into the lipid bilayer. Despite interacting with membranes, gladiolin on its own did not cause membrane rearrangements or damage in our AFM experiments (Fig. 5). This is consistent with its inability to inhibit fungal growth or reduce metabolic activity of mammalian cells when applied on its own.

Although gladiolin alone does not damage the membrane, it dramatically increases membrane damage in combination with AmpB. Membrane lipid rearrangements included thinning and formation of membrane pits, as well as thickening and the formation of pores leading to the destruction of the membrane. The areas of lipid rearrangement were bigger, and the changes occurred faster with the gladiolin/AmpB combination (within 10 minutes) compared with AmpB alone (within 60 min). We further show that another polyene antifungal, natamycin does not synergize with gladiolin (Fig. 3A through C) and does not cause increased membrane damage in combination with gladiolin (Fig. S4B). Together, these results support a mechanism of antifungal synergism between gladiolin and AmpB that results from increased membrane damage with the compound combination.

We found no evidence of gladiolin and AmpB interacting with each other in solution (Fig. S3A and B) and increasing the solubility of AmpB in the deoxycholate formulation (i.e., fungizone) did not elevate the degree of synergism with gladiolin (Fig. S3C). Together, these data argue that gladiolin does not simply solubilize AmpB. However, it remains possible that gladiolin and AmpB interact within membranes. Indeed, our SPR experiments show that gladiolin binds to membranes and changes the membrane-binding profile of AmpB to be biphasic and partially irreversible. While gladiolin on its own does not detectably damage the membrane in AFM experiments, the change in AmpB’s interaction with the membrane in the presence of gladiolin increases its ability to damage membranes, explaining the synergism between these two compounds.

Our study further provides insight into the MOAs of AmpB and how gladiolin potentiates these. Pore formation has been widely accepted as a mechanism responsible for the antifungal activity of AmpB. However, more recent work called this into question by showing that AmpB sequesters ergosterol as a “sponge” on the surface of the membrane rather than inserting into it to form pores (11, 12). In contrast, recent advanced imaging experiments using polarization-sensitive simulated Raman scattering are consistent with AmpB assembling into pores (14). Using AFM, we observed that AmpB causes both membrane thinning (pits), which, as shown before by others using neutron reflectometry, is caused by ergosterol extraction (41, 49). We further observed pore formation within areas of thickened lipid bilayers. The ability of gladiolin to enhance ergosterol extraction by AmpB is supported by the fact that, in AFM experiments, the areas of membrane thinning appeared faster in combination with gladiolin and the patches were larger (Fig. 5). With AFM, we also detected faster pore formation with gladiolin/AmpB combinations relative to AmpB alone. On a biophysical level, the two proposed MOAs for AmpB are not mutually exclusive, and our data support the notion that both mechanisms occur dynamically in fungal cell membranes and can be potentiated by compounds such as gladiolin. The finding that natamycin, which is unable to form membrane pores (13), does not synergize with gladiolin (Fig. 3C) supports the proposition that synergism of polyene compounds with gladiolin requires an enhancement of both MOAs (pore formation and ergosterol extraction), as observed for AmpB.

In conclusion, gladiolin exemplifies how natural products can be used as tools to understand the MOA and synergistically enhance the activity of current antifungal drugs. The ability of gladiolin to exacerbate membrane damage and synergistically reduce fungal growth without increasing the toxicity of AmpB against mammalian cells provides insights into how the activity/toxicity relationship of AmpB could be improved. This kind of potentiation strategies complement other efforts to reduce host toxicity and bioavailability, including but not limited to AmpB lipid and nanoparticle-based formulations and the synthesis of AmpB analogs with reduced binding to mammalian cholesterol (7, 15, 50). Given the scarcity of antifungal treatments, the growing resistance to both azoles and echinocandins, and the relatively small number of antifungal compounds in the development pipeline (51), it is important to take a multi-pronged approach to improve therapeutic options. Our study contributes to these efforts by characterizing a potentiator of AmpB.

## MATERIALS AND METHODS

### Fungal strains and media

All *Candida* and *Cryptococcus* strains are listed in Table S2 and were maintained on YPD plates (1% yeast extract, 2% peptone, 2% glucose, and 2% agar). For *Candida* precultures, single colonies were picked from YPD plates, inoculated into liquid YPD medium, and grown at 30°C overnight. All experiments were carried out in RPMI-1640 medium (R6504, Sigma) buffered with 3.5% MOPS and adjusted to pH 7.0 (hyphal growth) or pH 5.6 (yeast growth). For alternative C-sources, RPMI-1640 medium (R1383, Sigma) with 3.5% MOPS adjusted to pH7.0 was used.

### MIC, CFU, checkerboard, and filamentation experiments

Gladiolin was purified as described previously (27) and dissolved in 100% DMSO to achieve a stock concentration of 5 mg/mL. Gladiolin stocks were diluted in RPMI-1640 medium to twofold (MIC, CFU and filamentation experiments) or fourfold concentrated (checkerboard experiments). Fungal inocula were prepared as described in CLSI-M27. In brief, five colonies of fungal cultures were suspended in PBS and adjusted to an optical density (OD_600_) of 0.08 to 0.1. A volume of 100 µL of cell suspension was added to 9.9 mL RPMI-1640 medium to make the work solution. Fifty microliters of gladiolin (final concentration 0.25–16 µg/mL) and 50 µL of work solution were added into flat bottom 96-well plates (Nunc Microwell 167008, Thermo Scientific) and incubated at either 30°C or 37°C for 20 h. For CFU experiments, aliquots of the MIC assay were diluted appropriately, and 100 µL of cell suspension was plated onto YPD plates. After 2 days of incubation at 30°C, CFUs were counted. For synergism experiments, 25 µL of gladiolin concentrations, 25 µL of antifungal drugs (caspofungin, fluconazole, and amphotericin B), and 50 µL of work solution were mixed and incubated at 37°C for 20 or 48 h for *Cryptococcus*. Cell density at 600 nm was measured using a plate reader (Tecan, Spark 10M), or images were taken with an Olympus BX60 microscope at 40× magnification. Synergistic effects were evaluated using the fractional inhibitory concentration index (FICI) method, whereby MIC_A_ and MIC_B_ are defined as the minimal inhibitory concentrations of each drug alone and MIC_AC_ and MIC_BC_ are the corresponding MICs of both drugs in combination:


$$FICI=\frac{MIC_{AC}}{MIC_{A}}+\frac{MIC_{BC}}{MIC_{B}}$$

Drug interactions with FICI values of <0.5, >0.5 to <1, 1–4, or >4 are categorized as synergistic, additive, indifferent, and antagonistic, respectively (32, 33).

### Macrophage infection and live cell imaging experiments

Bone marrow-derived macrophages (BMDMs) were isolated and differentiated as we have previously described using femur and tibia bones of 6- to 8-week-old C57BL/6 mice (52). The medium for differentiation was RPMI-1640 medium supplemented with 12.5 mM HEPES, 20% L-cell conditioned medium, 15% fetal bovine serum, and 100 U/mL penicillin-streptomycin. Differentiation lasted for 6–8 days at 37°C and 5% CO_2_. Seeding of BMDMs was at 1 × 10^5^ cells/well in a 96-well plate, followed by incubation at 37°C and 5% CO_2_ overnight. After this step, macrophages were stained for 30 min with 1 µM CellTracker Green CMFDA (ThermoFisher) and then co-incubated with *C. albicans* strain SC5314 (MOI 3 yeast:1 macrophage). The phagocytosis step proceeded for 1 h, after which the remaining yeast cells were removed and warm BMDM medium with or without 16 µg/mL gladiolin or gladiolin/AmpB combinations was replenished. For imaging in the *C. albicans* experiments, 0.6 µM DRAQ7 (Abcam) was added as well, followed by the 96-well plate being put into an incubation chamber and time-lapse images acquired with a Leica AF6000 LX epifluorescence microscope every 30 min for up to 24 h using a Leica DMi8 Live Cell Imaging System and HC PL FLUOTAR L 20×/0.40 Dry PH1 CORR objective with bright field, GFP, and Y5 filters. For data analysis, the CellProfiler 2.1.1 software was used as we have previously described (53). DRAQ7 positive events were plotted using Prism 9 (Graphpad) software. For statistical analysis, a one-way ANOVA with Dunnett’s multiple comparisons test was used.

### Resazurin viability assay

BMDMs were seeded at 5 × 10^4^ cells/well in a 96-well plate in BMDM medium and incubated at 37°C and 5% CO_2_ overnight. The next day, 100 µL of twofold dilutions of gladiolin in BMDM medium was added to the cells and incubated at 37°C and 5% CO_2_ for 20 h followed by the addition of 10 µL 10× resazurin. Cells were further incubated at 37°C and 5% CO_2_ until reduction of resazurin was visible (2–4 h). Fluorescence (Ex./Em. 535/585) was measured using a plate reader (Tecan, Spark 10M).

### *Galleria mellonella* infection model

Before infection *Galleria mellonella* larvae (UK Waxworm Ltd) were decontaminated with 70% ethanol. Colonies of *C. albicans* were collected from YPD plates, resuspended in PBS, and adjusted to a cell density of 1 × 10^7^ cells/mL. Individual larvae were infected by injection of 10 µL of *Candida* suspension in the rear proleg with or without drug using an insulin syringe (BD Ultra-Fine). Larvae injected with 10 µL PBS served as uninfected control. Larvae were placed individually in wells of a 24-well plate, incubated at 37°C, and monitored for movement and discoloration over 7 days.

### Ventilator-associated pneumonia biofilm model

Biofilms were established on endotracheal tubes (ETT) (siliconized PVC, cuffed, 8 mm internal diameter, manufactured and supplied by IMS Euro) as described here (36). *C. albicans* precultures were grown in YPD at 37°C overnight. Cultures were adjusted to an OD of 0.05 in SVAM medium (36), and 0.5 mL of fungal suspension was added to ETT cuttings placed into 24-well plates. Plates were incubated for 48 h at 37°C and 5% CO_2_ before ETT cuttings were transferred to a fresh 24-well plate containing 0.5 mL SVAM medium with drug treatment. The plate was incubated for a further 24 h as before. The biofilm was dislodged in a sonicating water bath (Grant XUBA1) at 50 Hz for 15 min, resuspended and plated onto YPD plates for CFU counting. Synergistic drug interactions were verified as previously described (54) using the response-additivity model (55, 56).

### Rhodamine 6G efflux assay

Overnight cultures grown in YPD medium at 30°C were washed in PBS and adjusted to a cell density of 1 × 10^8^ cells/mL. Cells were incubated with 10 µg/mL R6G at 37°C for 1 h. Dye-loaded *Candida* cells were harvested and resuspended in PBS to 1 × 10^8^ cells/mL. Aliquots of 4 mL cell suspensions were treated with gladiolin and/or AmpB as indicated. Five-hundred microliters of aliquots were taken every 30 min for 2 h and centrifuged to remove the cells. The efflux of the dye into the supernatant was measured as absorbance at 520 nm using a plate reader (Tecan, Spark 10M).

### Liposome preparation

Ergosterol (Sigma-Aldrich) and 1-palmitoyl-2-oleoyl-glycero-3-phosphocholine (POPC) (Avanti Polar Lipids) were dissolved in chloroform at 2 µM. Dried lipid films were prepared by adding each lipid solutions to the bottom of clean, dried glass test tubes at a final volume of 500 µL at molar ratio of POPC (100%); POPC:ergosterol (90:10) and POPC:ergosterol (80:20). The organic solvent was evaporated with a gentle stream of dry N_2_ at 40°C. The residual organic solvent in dried lipid films was completely removed under high vacuum for 16 h. The dried lipid films were flushed with argon gas, sealed, and kept at −78°C until use. For the liposome preparation, 800 µL 10 mM HEPES, 150 mM NaCl, pH 7.2 (HBS), was added to each tube to hydrate the lipid films and vortex at high speed for 5 min. The resulting liposome suspensions about 1 mg/mL were incubated at 37°C for 1 h while shaking at 120 rpm/min, followed by bath-sonication for 30 min until full transparency. The clear liposome-HBS solutions were passed through a polycarbonate membrane (ATA scientific, Lucas Heights, Australia) with 100 nm pore diameter 31 times with AVESTIN Liposofast extruder (Avestin, Canada). The liposome size was analyzed by the Zetasizer NanoZS (Malvern Panalytical Ltd, UK).

### Surface plasmon resonance analysis

SPR experiments were carried out with a Biacore T100 analytical system on L1 sensor chip (S-series, Biacore, Uppsala, Sweden). Prior to the experiment, an L1 chip was docked and system was primed with 10 mM HEPES, 150 mM NaCl, pH 7.2 (HBS). The chip surface was pre-cleaned twice with 10 µL of 40 mM CHAPS at 5 µL/min followed by 10 µL of isopropanol containing 50 mM NaOH (vol:vol = 3:2) at 5 µL/min through all four flow cells. The chip surface was equilibrated in HBS running buffer at 10 µL/min for 20 min until no baseline drift. The liposome solution for SPR analysis was diluted to 0.2 mg/mL with HBS, and a solution of 100 mM CaCl_2_-HBS was added to reach a final concentration of 2 mM CaCl_2_-HBS. The diluted liposome solutions of each lipid composition in 2 mM CaCl_2_-HBS were then injected at 2 µL/min for 60 min. At the end of the liposome injection, the deposited lipid surfaces were pulse-rinsed twice with 30 mM EDTA-HBS at 30 µL/min for 1 min to remove Ca^2+^ and multi-lamellar structures from the lipid surface and to stabilize the baseline. The responses for all lipid compositions were 4,600–5,400RU. The interaction of gladiolin with each model lipid bilayers was examined at concentrations 0.5, 1, 2, 4, 8, and 16 µg/mL, where the 0.5, 1, and 2 µg/mL gladiolin in HBS contains 0.2% DMSO, and the 4, 8, and 16 µg/mL gladiolin were prepared in 0.4% DMSO-HBS. All binding experiments were carried out at 25°C. Each concentration of gladiolin, AmpB, or gladiolin + AmpB was injected at 30 µL/min with a total injection time of 200 s followed by a dissociation time of 400 s to give the sensorgrams shown in Fig. 4. The surface of L1 chip was regenerated by injecting twice with 10 µL of 40 mM CHAPS at 5 µL/min followed by 10 µL of isopropanol containing 50 mM NaOH (vol:vol = 3:2) at 5 µL/min. The sensorgrams for lipid deposition and each polyketide-membrane interaction were analyzed with BIA evaluation 4.0 software (Biacore, GE Health). Fitting the binding curves with two-state model for the kinetics resulted in poor fitting, and no kinetic constants can be obtained with confidence. Each of the binding curves was exported and plotted with SigmaPlot version 14.5 to illustrate the overall binding responses.

### Supported lipid bilayer formation

The supported lipid bilayers (SLBs) were prepared *ex situ* with vesicle adsorption-fusion methods. Liposome solution (1 mg/mL, 100 nm diameter) was diluted with HBS to 0.4 mg/mL, and 100 mM CaCl_2_-HBS was added to reach a final concentration of 4 mM Ca^2+^. Two hundred microliters of the liposome solution with 4 mM Ca^2+^ was added onto the surface of freshly cleaved muscovite mica (grade V-1, 12 mm diam) (Ted Pella Inc, CA, USA) glued to a parafilm-coated metal disk. The samples were placed in saturated humidity chambers and incubated at 30°C for 2–3 h in a programmable incubator. The SLBs were carefully and thoroughly rinsed with Ca^2+^-free HBS. The final SLBs on mica were kept under aqueous environment by adding 200 µL HBS to the surface and allowed to equilibrate to room temperature before imaging.

### Atomic force microscopy

The topography of the SLBs was characterized on FastScan Bio AFM (Bruker AXS, CA, USA) using PeakForce Mapping in Fluid in Nanomechanical Mapping. The instrument was controlled by NanoScope 9.1 software. A triangular ScanAsyst-Fluid+ probe (Bruker, CA, USA) with a nominal tip radius of 2 nm and a nominal spring constant of 0.7 N/m was used for imaging in fluid condition. The deflection sensitivity of the probe was calibrated on a sapphire reference sample in PeakForce QNM sample kit (Bruker, CA, USA). A mean deflection sensitivity of 23.5 nm/V obtained from three measurements was entered manually. The spring constant of 0.75 N/m was determined using thermal tuning on simple harmonic oscillator model in fluid. The tip radius was calibrated on a RS Ti roughness sample using Tip Qualification function in NanoScope Analysis software. The SLB samples were loaded to the scanner with a droplet method where the probe loaded onto the scanner was pre-wet with 30 µL HBS followed by engaging the sample. The SLBs were scanned with force setpoint manually maintained at 750 pN with the feedback gain automatically adjusted by software. The amplitude and frequency of peak force were set at 100 nm and 2 kHz, respectively. Prior to the addition of AmpB, natamycin, gladiolin, and mixtures, the POPC/ergosterol (80:20) SLBs were scanned at 1 kHz with 512 line-resolution at discrete area of 3 × 3, 5 × 5, and 10 × 10 µm in size, and the SLBs remain stable for at least 40 min during constant scanning. The real-time changes in the topography of POPC:ergosterol (80:20) induced by each drug and mixtures were tracked at a scan rate of 2 kHz for 120 min. The imaging size was 5 × 5 µm and with a line resolution of 256 and zoom-in images of varying dimensions were scanned at a line resolution of 512. The topographic images were analyzed with NanoScope Analysis software and processed in Gwyddion 2.51 software.

## SUPPLEMENTAL MATERIAL

Data set S1 mbio.02611-24-s0001.xlsx Numerical data used to make the figures.

Supplemental data mbio.02611-24-s0002.pdf Supplemental results and materials and methods for NMR, Figures S1-S4, and Tables S1 and S2.

Legends mbio.02611-24-s0003.pdf Supplemental legends.

Movie S1 mbio.02611-24-s0004.mp4 AFM time course of POPC:ergosterol model lipids with ratios of 80%:20% under control conditions.

Movie S2 mbio.02611-24-s0005.mp4 AFM time course of POPC:ergosterol model lipids with ratios of 80%:20% with 20 µg/ml gladiolin.

Movie S3 mbio.02611-24-s0006.mp4 AFM time course of POPC:ergosterol model lipids with ratios of 80%:20% with 20 µg/ml AmpB.

Movie S4 mbio.02611-24-s0007.mp4 AFM time course of POPC:ergosterol model lipids with ratios of 80%:20% with 10 µg/ml gladiolin and 10 µg/ml AmpB in combination.

Movie S5 mbio.02611-24-s0008.mp4 AFM time course of POPC:ergosterol model lipids with ratios of 80%:20% with 20 µg/ml natamycin.

Movie S6 mbio.02611-24-s0009.mp4 AFM time course of POPC:ergosterol model lipids with ratios of 80%:20% with 10 µg/ml gladiolin and 10 µg/ml natamycin in combination.

ASM does not own the copyrights to Supplemental Material that may be linked to, or accessed through, an article. The authors have granted ASM a non-exclusive, world-wide license to publish the Supplemental Material files. Please contact the corresponding author directly for reuse.

## References

[1] Denning DW. 2024. Global incidence and mortality of severe fungal disease. Lancet Infect Dis 24:e428–e438. doi:10.1016/S1473-3099(23)00692-8 38224705

[2] Hoenigl M, Seidel D, Sprute R, Cunha C, Oliverio M, Goldman GH, Ibrahim AS, Carvalho A. 2022. COVID-19-associated fungal infections. Nat Microbiol 7:1127–1140. doi:10.1038/s41564-022-01172-2 35918423 PMC9362108

[3] Rayens E, Norris KA. 2022. Prevalence and healthcare burden of fungal infections in the United States, 2018. Open Forum Infect Dis 9:ofab593. doi:10.1093/ofid/ofab593 35036461 PMC8754384

[4] Iyer KR, Revie NM, Fu C, Robbins N, Cowen LE. 2021. Treatment strategies for cryptococcal infection: challenges, advances and future outlook. Nat Rev Microbiol 19:454–466. doi:10.1038/s41579-021-00511-0 33558691 PMC7868659

[5] Lee Y, Robbins N, Cowen LE. 2023. Molecular mechanisms governing antifungal drug resistance. NPJ Antimicrob Resist 1:5. doi:10.1038/s44259-023-00007-2 38686214 PMC11057204

[6] Gow NAR, Johnson C, Berman J, Coste AT, Cuomo CA, Perlin DS, Bicanic T, Harrison TS, Wiederhold N, Bromley M, Chiller T, Edgar K. 2022. The importance of antimicrobial resistance in medical mycology. Nat Commun 13:5352. doi:10.1038/s41467-022-32249-5 36097014 PMC9466305

[7] Carolus H, Pierson S, Lagrou K, Van Dijck P. 2020. Amphotericin B and other polyenes-discovery, clinical use, mode of action and drug resistance. J Fungi (Basel) 6:321. doi:10.3390/jof6040321 33261213 PMC7724567

[8] Vincent BM, Lancaster AK, Scherz-Shouval R, Whitesell L, Lindquist S. 2013. Fitness trade-offs restrict the evolution of resistance to amphotericin B. PLoS Biol 11:e1001692. doi:10.1371/journal.pbio.1001692 24204207 PMC3812114

[9] Kamiński DM. 2014. Recent progress in the study of the interactions of amphotericin B with cholesterol and ergosterol in lipid environments. Eur Biophys J 43:453–467. doi:10.1007/s00249-014-0983-8 25173562 PMC4212203

[10] Kristanc L, Božič B, Jokhadar ŠZ, Dolenc MS, Gomišček G. 2019. The pore-forming action of polyenes: from model membranes to living organisms. Biochim Biophys Acta Biomembr 1861:418–430. doi:10.1016/j.bbamem.2018.11.006 30458121

[11] Gray KC, Palacios DS, Dailey I, Endo MM, Uno BE, Wilcock BC, Burke MD. 2012. Amphotericin primarily kills yeast by simply binding ergosterol. Proc Natl Acad Sci U S A 109:2234–2239. doi:10.1073/pnas.1117280109 22308411 PMC3289339

[12] Anderson TM, Clay MC, Cioffi AG, Diaz KA, Hisao GS, Tuttle MD, Nieuwkoop AJ, Comellas G, Maryum N, Wang S, Uno BE, Wildeman EL, Gonen T, Rienstra CM, Burke MD. 2014. Amphotericin forms an extramembranous and fungicidal sterol sponge. Nat Chem Biol 10:400–406. doi:10.1038/nchembio.1496 24681535 PMC3992202

[13] Welscher Y te, Napel H ten, Balagué MM, Souza CM, Riezman H, de Kruijff B, Breukink E. 2008. Natamycin blocks fungal growth by binding specifically to ergosterol without permeabilizing the membrane. J Biol Chem 283:6393–6401. doi:10.1074/jbc.M707821200 18165687

[14] Dong PT, Zong C, Dagher Z, Hui J, Li J, Zhan Y, Zhang M, Mansour MK, Cheng JX. 2021. Polarization-sensitive stimulated Raman scattering imaging resolves amphotericin B orientation in Candida membrane. Sci Adv 7:eabd5230. doi:10.1126/sciadv.abd5230 33523971 PMC7787481

[15] Maji A, Soutar CP, Zhang J, Lewandowska A, Uno BE, Yan S, Shelke Y, Murhade G, Nimerovsky E, Borcik CG, et al.. 2023. Tuning sterol extraction kinetics yields a renal-sparing polyene antifungal. Nature 623:1079–1085. doi:10.1038/s41586-023-06710-4 37938782 PMC10883201

[16] Laniado-Laborín R, Cabrales-Vargas MN. 2009. Amphotericin B: side effects and toxicity. Rev Iberoam Micol 26:223–227. doi:10.1016/j.riam.2009.06.003 19836985

[17] WHO. 2022. Guidelines for diagnosing, preventing and managing cryptococcal disease among adults, adolescents and children living with HIV 35797432

[18] Pappas PG, Kauffman CA, Andes DR, Clancy CJ, Marr KA, Ostrosky-Zeichner L, Reboli AC, Schuster MG, Vazquez JA, Walsh TJ, Zaoutis TE, Sobel JD. 2016. Clinical practice guideline for the management of candidiasis: 2016 update by the infectious diseases society of America. Clin Infect Dis 62:e1–e50. doi:10.1093/cid/civ933 26679628 PMC4725385

[19] Verweij PE, Ananda-Rajah M, Andes D, Arendrup MC, Brüggemann RJ, Chowdhary A, Cornely OA, Denning DW, Groll AH, Izumikawa K, Kullberg BJ, Lagrou K, Maertens J, Meis JF, Newton P, Page I, Seyedmousavi S, Sheppard DC, Viscoli C, Warris A, Donnelly JP. 2015. International expert opinion on the management of infection caused by azole-resistant Aspergillus fumigatus. Drug Resist Updat 21–22:30–40. doi:10.1016/j.drup.2015.08.001 26282594

[20] CDC. Treatment and management of C. auris infections and colonization

[21] WHO. 2022. WHO fungal priority pathogens list to guide research development and public health action

[22] Dróżdż A, Kubera D, Sławińska-Brych A, Matwijczuk A, Ślusarczyk L, Czernel G, Karcz D, Olender A, Bogut A, Pietrzak D, Dąbrowski W, Stepulak A, Wójcik-Załuska A, Gagoś M. 2023. Synergistic antifungal interactions between antibiotic amphotericin B and selected 1,3,4-thiadiazole derivatives, determined by microbiological, cytochemical, and molecular spectroscopic studies. Int J Mol Sci 24:3430. doi:10.3390/ijms24043430 36834848 PMC9966784

[23] Chudzik B, Bonio K, Dabrowski W, Pietrzak D, Niewiadomy A, Olender A, Malodobry K, Gagoś M. 2019. Synergistic antifungal interactions of amphotericin B with 4-(5-methyl-1,3,4-thiadiazole-2-yl) benzene-1,3-diol. Sci Rep 9:12945. doi:10.1038/s41598-019-49425-1 31506532 PMC6737028

[24] Escrig JI, Hahn HJ, Debnath A. 2020. Activity of auranofin against multiple genotypes of Naegleria fowleri and its synergistic effect with amphotericin B in vitro. ACS Chem Neurosci 11:2464–2471. doi:10.1021/acschemneuro.0c00165 32392039 PMC7442663

[25] Fernandes KE, Payne RJ, Carter DA. 2020. Lactoferrin-derived peptide lactofungin is potently synergistic with amphotericin B. Antimicrob Agents Chemother 64:e00842-20. doi:10.1128/AAC.00842-20 32690642 PMC7508585

[26] Grela E, Stączek S, Nowak M, Pawlikowska-Pawlega B, Zdybicka-Barabas A, Janik S, Cytryńska M, Grudzinski W, Gruszecki WI, Luchowski R. 2023. Enhanced antifungal activity of amphotericin B bound to albumin: a "Trojan Horse" effect of the protein. J Phys Chem B 127:3632–3640. doi:10.1021/acs.jpcb.3c01168 37071547 PMC10150355

[27] Song L, Jenner M, Masschelein J, Jones C, Bull MJ, Harris SR, Hartkoorn RC, Vocat A, Romero-Canelon I, Coupland P, Webster G, Dunn M, Weiser R, Paisey C, Cole ST, Parkhill J, Mahenthiralingam E, Challis GL. 2017. Discovery and biosynthesis of gladiolin: a Burkholderia gladioli antibiotic with promising activity against Mycobacterium tuberculosis. J Am Chem Soc 139:7974–7981. doi:10.1021/jacs.7b03382 28528545

[28] Leclair LW, Hogan DA. 2010. Mixed bacterial-fungal infections in the CF respiratory tract. Med Mycol 48:S125–S132. doi:10.3109/13693786.2010.521522 21067324

[29] Kerr J. 1994. Inhibition of fungal growth by Pseudomonas aeruginosa and Pseudomonas cepacia isolated from patients with cystic fibrosis. J Infect 28:305–310. doi:10.1016/s0163-4453(94)91943-7 7522262

[30] Boon C, Deng Y, Wang LH, He Y, Xu JL, Fan Y, Pan SQ, Zhang LH. 2008. A novel DSF-like signal from Burkholderia cenocepacia interferes with Candida albicans morphological transition. ISME J 2:27–36. doi:10.1038/ismej.2007.76 18049456

[31] Hirakawa MP, Martinez DA, Sakthikumar S, Anderson MZ, Berlin A, Gujja S, Zeng Q, Zisson E, Wang JM, Greenberg JM, Berman J, Bennett RJ, Cuomo CA. 2015. Genetic and phenotypic intra-species variation in Candida albicans. Genome Res 25:413–425. doi:10.1101/gr.174623.114 25504520 PMC4352881

[32] Meletiadis J, Pournaras S, Roilides E, Walsh TJ. 2010. Defining fractional inhibitory concentration index cutoffs for additive interactions based on self-drug additive combinations, Monte Carlo simulation analysis, and in vitro-in vivo correlation data for antifungal drug combinations against Aspergillus fumigatus. Antimicrob Agents Chemother 54:602–609. doi:10.1128/AAC.00999-09 19995928 PMC2812160

[33] Odds FC. 2003. Synergy, antagonism, and what the chequerboard puts between them. J Antimicrob Chemother 52:1. doi:10.1093/jac/dkg301 12805255

[34] Liu J, Yu YT, Xu CH, Chen DC. 2020. Candida colonization in the respiratory tract: what is the significance? Front Med (Lausanne) 7:598037. doi:10.3389/fmed.2020.598037 33614672 PMC7889970

[35] Pendleton KM, Huffnagle GB, Dickson RP. 2017. The significance of Candida in the human respiratory tract: our evolving understanding. Pathog Dis 75:ftx029. doi:10.1093/femspd/ftx029 28423168 PMC6433300

[36] Walsh D, Parmenter C, Bakker SE, Lithgow T, Traven A, Harrison F. 2024. A new model of endotracheal tube biofilm identifies combinations of matrix-degrading enzymes and antimicrobials able to eradicate biofilms of pathogens that cause ventilator-associated pneumonia. Microbiol (Reading) 170. doi:10.1099/mic.0.001480 PMC11541551 39088248

[37] Austermeier S, Kasper L, Westman J, Gresnigt MS. 2020. I want to break free – macrophage strategies to recognize and kill Candida albicans, and fungal counter-strategies to escape. Curr Opin Microbiol 58:15–23. doi:10.1016/j.mib.2020.05.007 32599492

[38] Mouri R, Konoki K, Matsumori N, Oishi T, Murata M. 2008. Complex formation of amphotericin B in sterol-containing membranes as evidenced by surface plasmon resonance. Biochemistry 47:7807–7815. doi:10.1021/bi800334p 18597487

[39] Oka M, Kamimori H. 2013. Lipid membrane-binding properties of amphotericin B deoxycholate (Fungizone) using surface plasmon resonance. Anal Sci 29:697–702. doi:10.2116/analsci.29.697 23842411

[40] Onishi M, Kamimori H. 2013. High-throughput and sensitive assay for amphotericin B interaction with lipid membrane on the model membrane systems by surface plasmon resonance. Biol Pharm Bull 36:658–663. doi:10.1248/bpb.b12-01020 23546296

[41] Delhom R, Nelson A, Laux V, Haertlein M, Knecht W, Fragneto G, Wacklin-Knecht HP. 2020. The antifungal mechanism of amphotericin B elucidated in ergosterol and cholesterol-containing membranes using neutron reflectometry. Nanomater (Basel) 10:2439. doi:10.3390/nano10122439 PMC7762259 33291326

[42] Uchida R, Kondo A, Yagi A, Nonaka K, Masuma R, Kobayashi K, Tomoda H. 2019. Simpotentin, a new potentiator of amphotericin B activity against Candida albicans, produced by Simplicillium minatense FKI-4981. J Antibiot (Tokyo) 72:134–140. doi:10.1038/s41429-018-0128-x 30532035

[43] Fukuda T, Nagai K, Yagi A, Kobayashi K, Uchida R, Yasuhara T, Tomoda H. 2019. Nectriatide, a potentiator of amphotericin B activity from Nectriaceae sp. BF-0114. J Nat Prod 82:2673–2681. doi:10.1021/acs.jnatprod.8b01056 31498627

[44] Irschik H, Schummer D, Höfle G, Reichenbach H, Steinmetz H, Jansen R. 2007. Etnangien, a macrolide-polyene antibiotic from Sorangium cellulosum that inhibits nucleic acid polymerases. J Nat Prod 70:1060–1063. doi:10.1021/np070115h 17547459

[45] Mozsolits H, Wirth HJ, Werkmeister J, Aguilar MI. 2001. Analysis of antimicrobial peptide interactions with hybrid bilayer membrane systems using surface plasmon resonance. Biochim Biophys Acta 1512:64–76. doi:10.1016/s0005-2736(01)00303-0 11334625

[46] Hong Q, Gutierrez-Aguirre I, Barlic A, Malovrh P, Kristan K, Podlesek Z, Macek P, Turk D, Gonzalez-Manas JM, Lakey JH, Anderluh G. 2002. Two-step membrane binding by Equinatoxin II, a pore-forming toxin from the sea anemone, involves an exposed aromatic cluster and a flexible helix. J Biol Chem 277:41916–41924. doi:10.1074/jbc.M204625200 12198118

[47] Gaidukov L, Fish A, Mor A. 2003. Analysis of membrane-binding properties of dermaseptin analogues: relationships between binding and cytotoxicity. Biochemistry 42:12866–12874. doi:10.1021/bi034514x 14596600

[48] Hall K, Lee TH, Mechler AI, Swann MJ, Aguilar MI. 2014. Real-time measurement of membrane conformational states induced by antimicrobial peptides: balance between recovery and lysis. Sci Rep 4:5479. doi:10.1038/srep05479 24969959 PMC4073255

[49] de Ghellinck A, Fragneto G, Laux V, Haertlein M, Jouhet J, Sferrazza M, Wacklin H. 2015. Lipid polyunsaturation determines the extent of membrane structural changes induced by Amphotericin B in Pichia pastoris yeast. Biochim Biophys Acta 1848:2317–2325. doi:10.1016/j.bbamem.2015.06.006 26055896

[50] Aigner M, Lass-Flörl C. 2020. Encochleated amphotericin B: is the oral availability of amphotericin B finally reached? J Fungi (Basel) 6:66. doi:10.3390/jof6020066 32443486 PMC7344640

[51] Hoenigl M, Sprute R, Egger M, Arastehfar A, Cornely OA, Krause R, Lass-Flörl C, Prattes J, Spec A, Thompson GR 3rd, Wiederhold N, Jenks JD. 2021. The antifungal pipeline: fosmanogepix, ibrexafungerp, olorofim, opelconazole, and rezafungin. Drugs (Abingdon Engl) 81:1703–1729. doi:10.1007/s40265-021-01611-0 PMC8501344 34626339

[52] Tucey TM, Verma J, Harrison PF, Snelgrove SL, Lo TL, Scherer AK, Barugahare AA, Powell DR, Wheeler RT, Hickey MJ, Beilharz TH, Naderer T, Traven A. 2018. Glucose homeostasis is important for immune cell viability during Candida challenge and host survival of systemic fungal infection. Cell Metab 27:988–1006. doi:10.1016/j.cmet.2018.03.019 29719235 PMC6709535

[53] Olivier FAB, Hilsenstein V, Weerasinghe H, Weir A, Hughes S, Crawford S, Vince JE, Hickey MJ, Traven A. 2022. The escape of Candida albicans from macrophages is enabled by the fungal toxin candidalysin and two host cell death pathways. Cell Rep 40:111374. doi:10.1016/j.celrep.2022.111374 36130496

[54] Harrison F, Blower A, de Wolf C, Connelly E. 2023. Sweet and sour synergy: exploring the antibacterial and antibiofilm activity of acetic acid and vinegar combined with medical-grade honeys. Microbiol (Reading) 169:001351. doi:10.1099/mic.0.001351 PMC10433418 37435775

[55] Slinker BK. 1998. The statistics of synergism. J Mol Cell Cardiol 30:723–731. doi:10.1006/jmcc.1998.0655 9602421

[56] Duarte D, Vale N. 2022. Evaluation of synergism in drug combinations and reference models for future orientations in oncology. Curr Res Pharmacol Drug Discov 3:100110. doi:10.1016/j.crphar.2022.100110 35620200 PMC9127325

