# Supplementary material for: Gladiolin produced by pathogenic Burkholderia synergizes with amphotericin B through membrane lipid rearrangements
Source: mBio. 2024 Oct 18;15(11):e02611-24. doi: 10.1128/mbio.02611-24 (PMC11559049; doi:10.1128/mbio.02611-24)
Supplement: Supplemental data — Supplemental results and materials and methods for NMR, Figures S1-S4, and Tables S1 and S2. [file mbio.02611-24-s0002.pdf]

^{1}H and DOSY NMR results. ^{1}H NMR spectroscopy was performed on each sample using solvent suppression to reduce the residual water peak in the amphotericin B deoxycholate spectra and to reduce the residual DMSO peak in the spectra of the gladiolin and mixed samples. The ^{1}H NMR spectra (Fig S5) show that there is significant overlap between many of the signals of amphotericin B deoxycholate and gladiolin. Gladiolin has signals between 5 and 6 ppm which do not significantly overlap with amphotericin B deoxycholate and may be diagnostic in the later diffusion experiments, while amphotericin B deoxycholate has a singlet at 0.61 ppm which may be diagnostic. The optimum gradient pulse for diffusion NMR experiments was determined on the specific samples utilizing the one-dimensional version of the experiment. The optimized pseudo-two-dimensional bipolar gradient pulse stimulated echo experiments were performed on the three samples to determine the diffusion coefficients. The diffusion data were processed as diffusion-ordered spectra (DOSY) in order to visualize the diffusion coefficients. Amphotericin B deoxycholate (Fig S5, green) had a measured diffusion coefficient D of 3.3×10^{-10} m^2s^{-1} at 25°C and gladiolin (Fig S5, red) had a measured diffusion coefficient D of 2.0×10^{-10} m^2s^{-1} at 25°C, both in 1 mg/mL PBS solution. Only one diffusion coefficient is observed for all signals of amphotericin B deoxycholate suggesting that the two molecules behave as one species in solution or, less likely, they exist as separate species in solution which have the same diffusion coefficient. The DOSY plots show log(D) on the y-axis, therefore amphotericin B deoxycholate and gladiolin display as -9.48 and -9.69, respectively. As demonstrated by the Stokes-Einstein equation,

$$D = \frac{k \cdot T}{6\pi\eta r} \qquad [1]$$

the diffusion coefficients D are inversely proportional to viscosity η. Due to the limitations of quickly measuring viscosity on very small volumes of solution, concentration is used as a proxy for viscosity. The individual diffusion data for amphotericin B deoxycholate and gladiolin were collected on 1 mg/mL solutions while the mixed solution had a final concentration of 1 mg/mL, i.e. 0.5 mg/mL of each component. The Stokes-Einstein equation [1] also shows that the diffusion coefficient D is inversely proportional to hydrodynamic radius r. The value of r cannot be solved directly without an accurate value for viscosity, therefore utilizing the assumptions above for viscosity and at constant temperature, we assume the relationship where all other values are constant across the various measurements in this series. The log values of the measured diffusion coefficients for amphotericin B deoxycholate and for gladiolin are -9.48 and -9.69, respectively. When the DOSY spectrum (Fig S3) for the mixed sample is analyzed, it can be seen that there are signals at log(D) of -9.48 and -9.69, and spreading between these two extremes. This is an indication that where the ^{1}H chemical shifts are well separated, two different values for D can be calculated but where they overlap the average is returned. There are no correlations observed at smaller values of D which would be expected if the amphotericin B deoxycholate and gladiolin existed in association resulting in an increased hydrodynamic radius.

Material and Methods

NMR analysis. Stock solution of amphotericin B deoxycholate and gladiolin were prepared by dissolving drug in PBS/D_2O to a concentration of 1 mg/mL. NMR diffusion ordered spectroscopy (DOSY) and diffusion coefficients were determined using the pulsed field gradients spin echo method utilizing a Bruker Av400 NMR spectrometer equipped with a 5 mm broadband BB H/D probe operating at 400.13 MHz for 1H with a 5.35 G/mm z gradient. Bruker software TopSpin 3.5.7 was used for data acquisition and TopSpin 4.2.0 and Dynamics Center 2.8.1 were used for processing. Samples were maintained at 25°C ± 0.1°C and at least 30 minutes was allowed for the sample to reach thermal equilibrium before any measurements were made. The NMR data were measured with a bipolar gradient pulse stimulated echo sequence with one spoil gradient incorporating solvent suppression using the 3-9-19 pulse sequence. The diffusion time Δ was 50 ms and the gradient pulse length was 1.8 ms making a total gradient length δ of 3.6 ms. Gradient pulses were smoothed square chirp shape. Experiments were performed as pseudo 2D data collections with a linear variation of the gradient from 2 to 95% of maximum intensity in 32 steps. The data were processed and the peak areas I were used to fit the equation

$$I = I_0 e^{-D2\pi\gamma\delta^{1/2}\Delta - \delta 3\times10^4} \qquad [2]$$

to determine the diffusion coefficient D as single component fits. The Stokes-Einstein equation [1] is applicable to the diffusion of spherical particles through a liquid with low Reynolds number (laminar, or non-turbulent, flow). Solving the equation for r gives an indicative hydrodynamic radius, assuming the particle to be spherical, and also assuming the solution viscosity to be that of the neat solvent at 25°C.

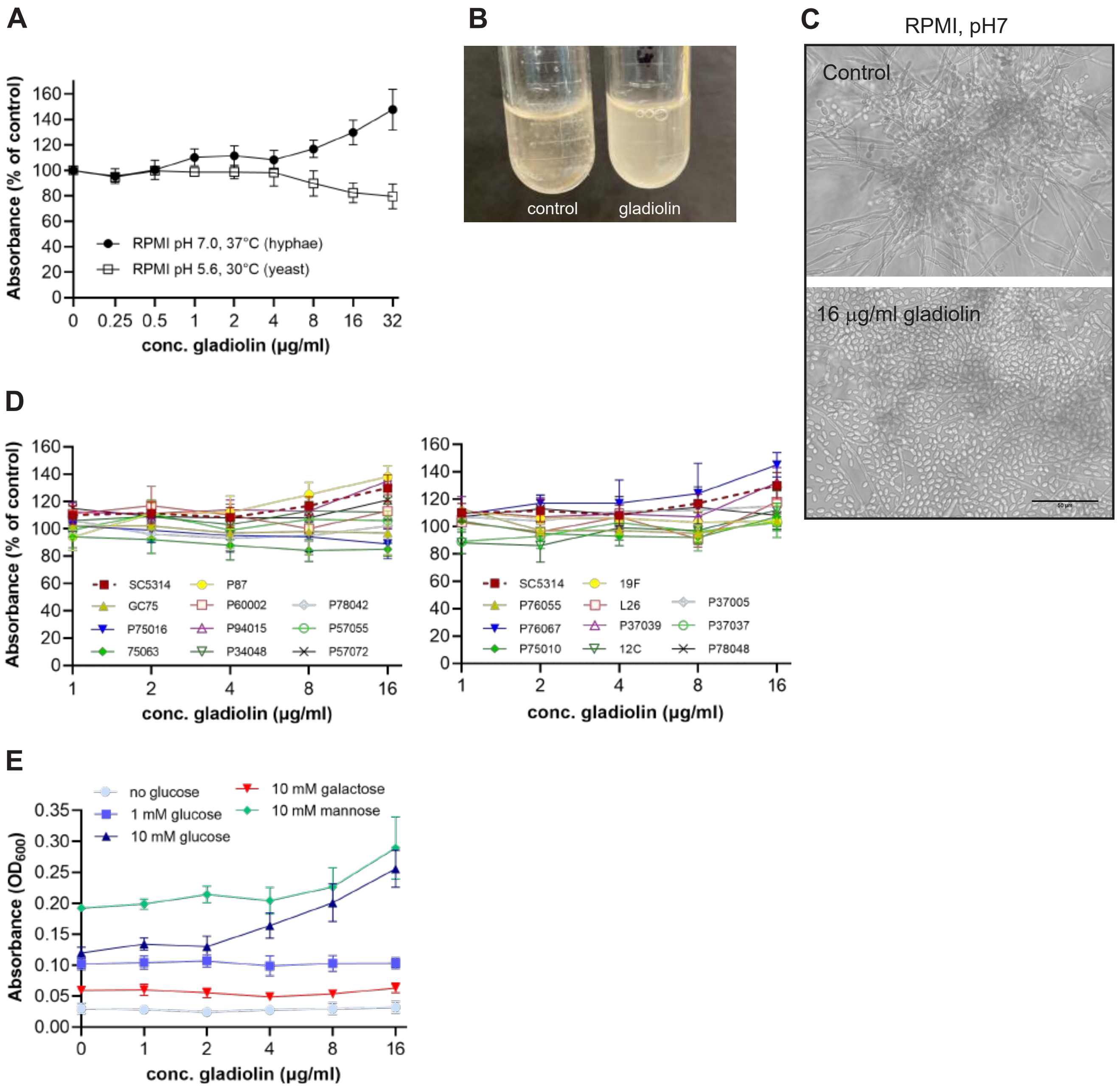

Fig. S1. Gladiolin does not cause growth inhibition but alters cell morphology of *C. albicans* A) Growth of *C. albicans* in either hyphal (RPMI, pH 7.0 at 37°C) or yeast conditions (RPMI, pH 5.6 at 30°C). Shown is the % absorbance (OD_{600}) of gladiolin-treated cultures relative to untreated samples (mean ± SEM; n = 3 to 6). B) The effects of gladiolin on the morphology of *C. albicans*. Cultures were grown in RPMI, pH 7.0 at 37°C in the presence or absence of 16 μg/ml gladiolin. Images of liquid cultures were taken after 20 hours of growth. C) Microscopy images of *C. albicans* cultures grown in RPMI, pH 7.0 with or without gladiolin. Images were taken after 20 hours of incubation at 37°C at a 40x magnification. Images were cropped and adjusted for brightness. The scale bar is 50 μm. D) *C. albicans* clinical isolates were grown in RPMI, pH 7.0 at 37°C. OD_{600} was measured after 20 hours. The data shown are the mean ± SEM (n = 3). For comparison data from reference strain SC5314 (panel A) were plotted on both graphs. E) *C. albicans* cultures were grown in RPMI, pH 7.0 supplemented with the indicated carbon sources at 37°C. Cell density at 600nm was measured after 20 hours. The data shown are the mean ± SEM (n = 4).

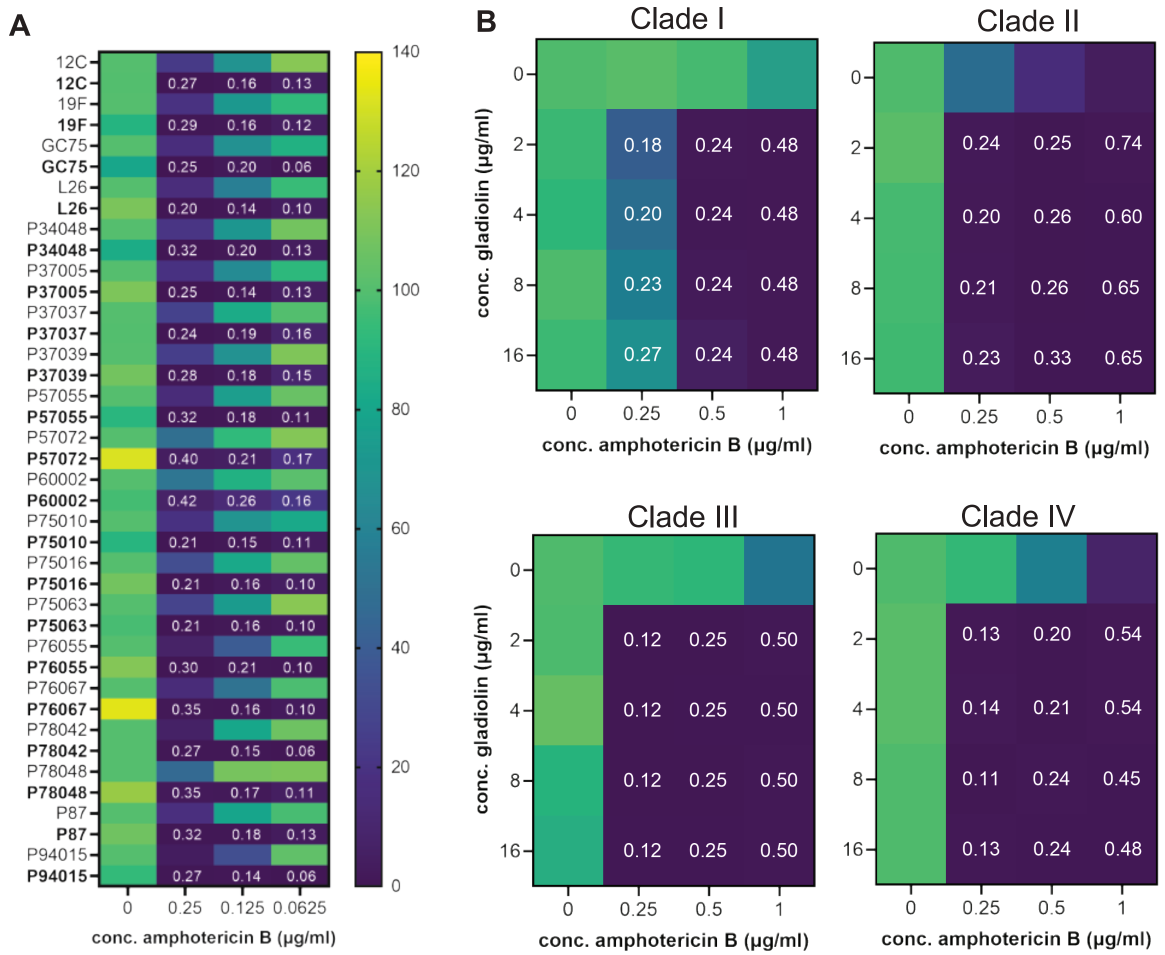

Fig. S2. Gladiolin potentiates AmpB against a compendium of diverse fungal clinical strains. A) Heatmap of gladiolin/AmpB checkerboard assays. A panel of clinical *C. albicans* isolates was grown in presence of 0.0625, 0.125 and 0.25 μg/ml AmpB alone or in combination with 4 μg/ml gladiolin (highlighted in bold) and growth was measured after 20 hours at 37°C. B) Heatmaps of gladiolin/AmpB checkerboard assays for *C. auris* isolates belonging to distinct genetic clades. Checkerboard assays were performed as described above. The color scale shows the percentage of survival in comparison to untreated control. The fractional inhibitory concentration index (FICI) is indicated as white numbers and is defined as < 0.5 (synergistic), > 0.5 < 1 (additive), 1 – 4 (indifferent) and > 4 (antagonistic). Heatmaps and FICIs were derived from mean values of 2 - 3 biological repeats.

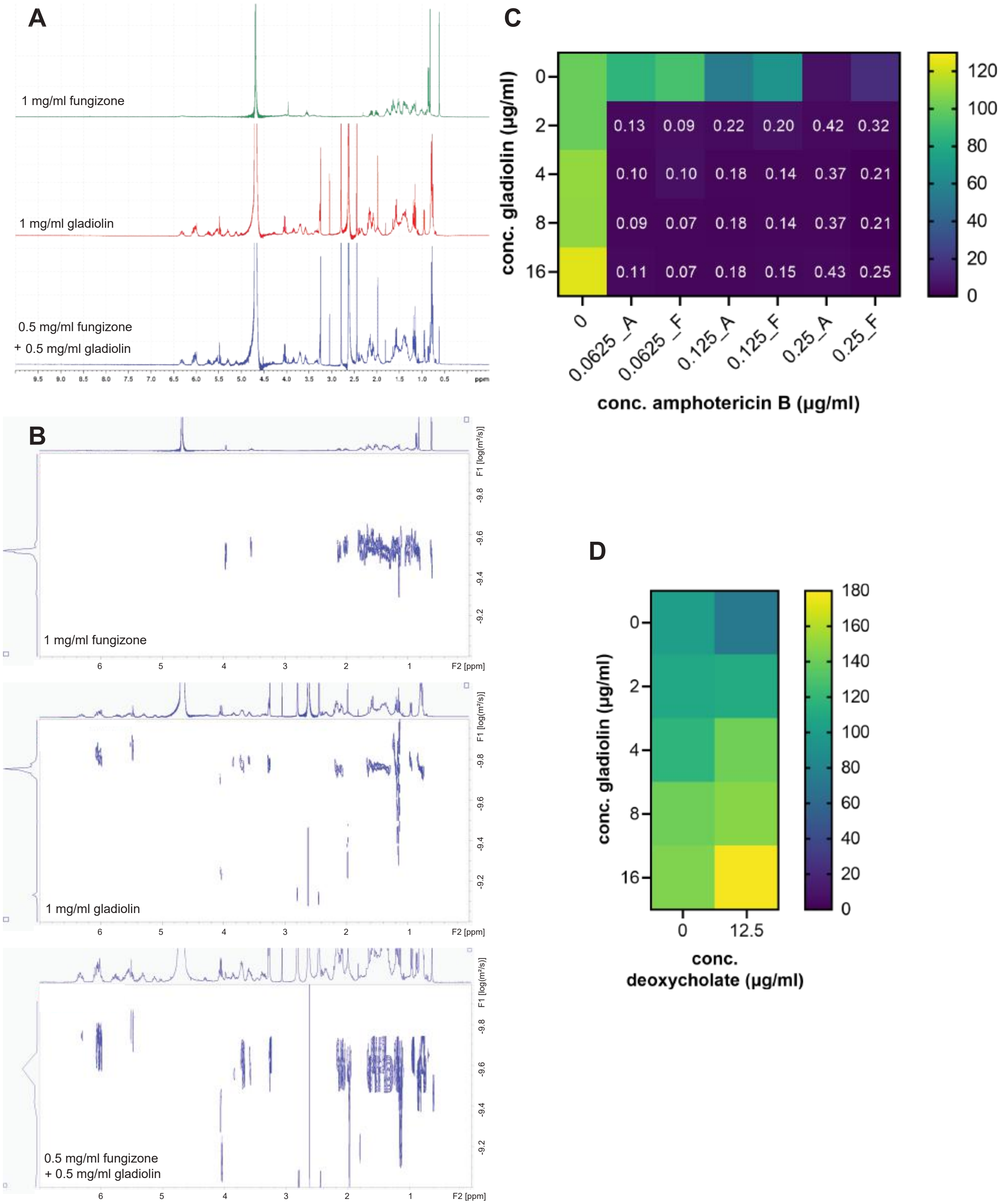

Fig. S3. Gladiolin does not physically interact with AmpB in solution or acts as a detergent to solubilize AmpB for improved antifungal efficacy. A) ^{1}H NMR spectra of fungizone (1 mg/mL in PBS/D_2O), gladiolin (1 mg/mL in PBS/D_2O), and fungizone and gladiolin in combination (each 0.5 mg/mL in PBS/D_2O); water 4.7 ppm, DMSO 2.61 ppm. B) DOSY NMR spectra of fungizone (1 mg/mL in PBS/D_2O), gladiolin (1 mg/mL in PBS/D_2O) and fungizone and gladiolin in combination (each 0.5 mg/mL in PBS/D_2O). C) Heatmap of checkerboard assays of gladiolin in combination with AmpB (A) or fungizone (F) assayed side-by-side. *C. albicans* cultures were grown in RPMI, pH 7.0 and cell density at 600nm was measured after 20 hours of growth at 37°C. The color scale shows the percentage of survival compared to untreated control. The fractional inhibitory concentration index (FICI) is indicated as white numbers and is defined as < 0.5 (synergistic), > 0.5 < 1 (additive), 1 – 4 (indifferent) and > 4 (antagonistic). The Heatmap and FICIs were generated from mean values of three biological repeats. D) Heatmap of checkerboard assays of gladiolin in combination with deoxycholate. The assay was carried out as in C. The heatmap was generated from mean values of two biological repeats. A non-interactive response for all tested combinations was confirmed via the Response-Additivity model.

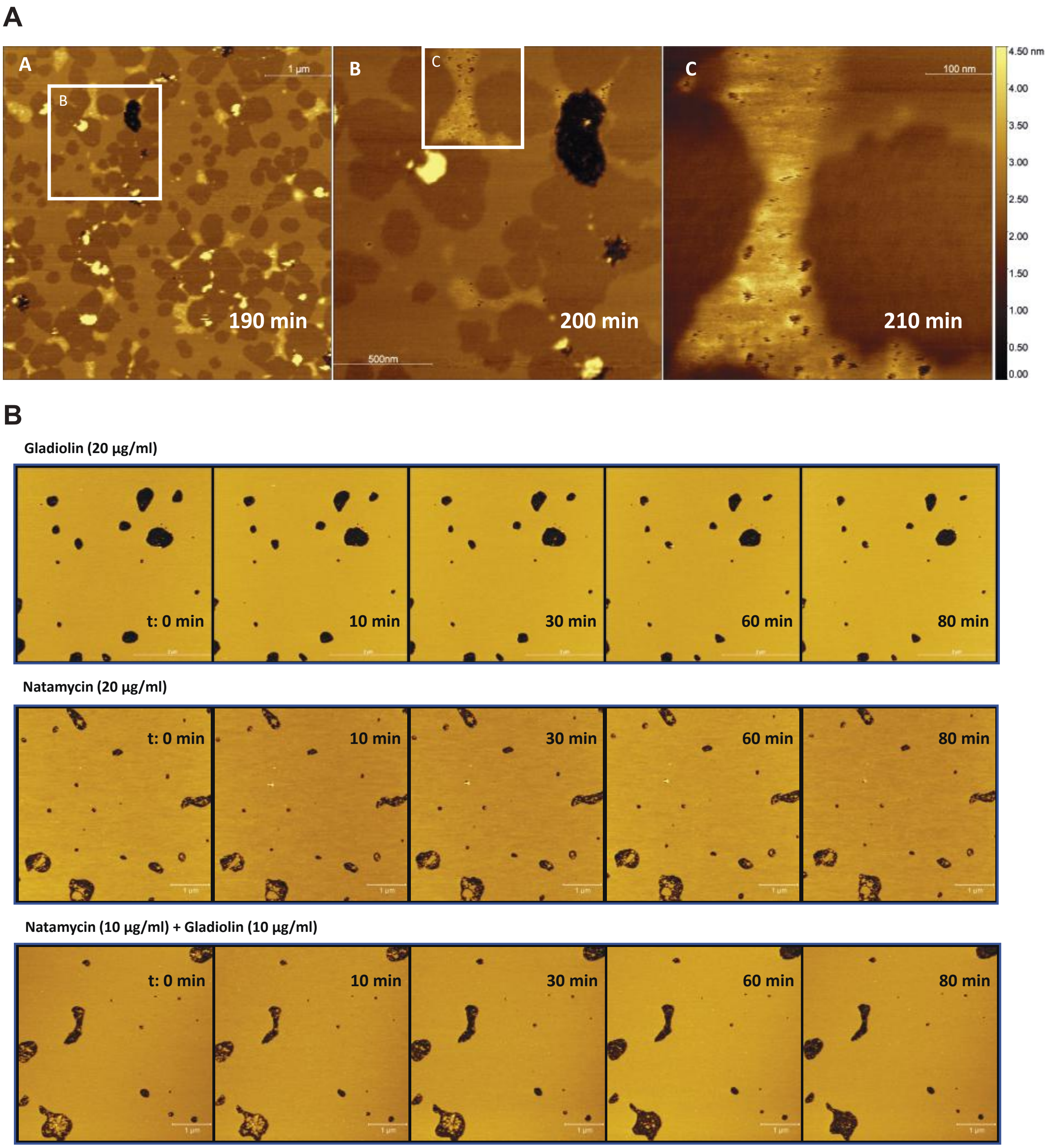

Fig. S4. Topographical changes of POPC:ergosterol supported lipid bilayers with gladiolin/polyene combinations. A) Magnifications of supported lipid bilayers (SLBs) treated with 10 μg/ml gladiolin and 10 μg/ml AmpB. Images are zoomed-in sections taken at 190 to 200 minutes. The color scale shows depth of the lipid bilayer. B) Time course of the topographical changes of POPC/ergosterol SLBs treated with gladiolin, natamycin or their combination. SLBs are treated with either gladiolin (20 μg/ml) or natamycin (20 μg/ml) alone or in combination at 10 μg/ml each.

Table S1. FICIs of gladiolin/AmpB combination for the *C. albicans* clinical isolates tested in Figure S2.

Strain name	2 ug/ml gladiolin			8 ug/ml gladiolin			16 ug/ml gladiolin		
	**0.0625 AmpB**	**0.125 AmpB**	**0.25 AmpB**	**0.0625 AmpB**	**0.125 AmpB**	**0.25 AmpB**	**0.0625 AmpB**	**0.125 AmpB**	**0.25 AmpB**
12C	0.28	0.17	0.33	0.24	0.16	0.27	0.21	0.17	0.27
19F	0.25	0.17	0.33	0.12	0.17	0.26	0.12	0.18	0.32
GC75	0.15	0.21	0.25	0.07	0.23	0.29	0.09	0.25	0.32
L26	0.22	0.15	0.23	0.29	0.17	0.25	0.37	0.16	0.28
P34048	0.28	0.20	0.32	0.11	0.19	0.26	0.14	0.20	0.32
P37005	0.27	0.16	0.15	0.13	0.17	0.31	0.70	0.17	0.25
P37037	0.32	0.19	0.25	0.16	0.18	0.34	0.17	0.21	0.38
P37039	0.32	0.18	0.25	0.17	0.18	0.27	0.17	0.21	0.31
P57055	0.22	0.16	0.34	0.09	0.16	0.32	0.10	0.17	0.29
P57072	0.34	0.20	0.61	0.14	0.20	0.28	0.15	0.22	0.24
P60002	0.34	0.26	0.19	0.17	0.28	0.20	0.17	0.27	0.21
P75010	0.27	0.14	0.19	0.10	0.14	0.27	0.14	0.17	0.27
P75016	0.34	0.20	0.61	0.14	0.20	0.28	0.15	0.22	0.24
P75063	0.19	0.15	0.25	0.10	0.14	0.33	0.12	0.18	0.28
P76055	0.21	0.12	0.30	0.09	0.13	0.36	0.12	0.17	0.39
P76067	0.23	0.15	0.31	0.10	0.16	0.27	0.10	0.18	0.33
P78042	0.14	0.16	0.31	0.07	0.08	0.33	0.09	0.15	0.29
P78048	0.21	0.15	0.30	0.11	0.16	0.21	0.10	0.11	0.25
P87	0.28	0.20	0.35	0.14	0.22	0.28	0.13	0.22	0.27
P94015	0.11	0.14	0.16	0.07	0.13	0.26	0.08	0.14	0.30

Table S2. Fungal strains used in the study.

Strain	Name	Description	Genotype	Species	Source
YCAT229	SC5314	Clinical isolate, from patient with "generalised Candida infection"	*C. albicans*	Clade 1	Traven Lab
YCAT793	GC75	Clinical isolate, healthy individual, oral sample	*C. albicans*	Clade 4	BEI Resources, NIAID, NIH: Candida albicans, Strain GC75, NR-29452.
YCAT794	P75016	Clinical isolate, BSI	*C. albicans*	Clade 4	BEI Resources, NIAID, NIH: Candida albicans, Strain P75016, NR-29438.
YCAT795	P75063	Clinical isolate, BSI	*C. albicans*	Clade 4	BEI Resources, NIAID, NIH: Candida albicans, Strain P75063, NR-29440.
YCAT796	P87	Clinical isolate, HIV+, oral sample	*C. albicans*	Clade 4	BEI Resources, NIAID, NIH: Candida albicans, Strain P87, NR-29453.
YCAT797	P60002	Clinical isolate, BSI	*C. albicans*	Clade 8	BEI Resources, NIAID, NIH: Candida albicans, Strain P60002, NR-29448.
YCAT798	P94015	Clinical isolate, BSI	*C. albicans*	Clade 6	BEI Resources, NIAID, NIH: Candida albicans, Strain P94015, NR-29446.
YCAT799	P34048	Clinical isolate, BSI	*C. albicans*	Clade 3	BEI Resources, NIAID, NIH: Candida albicans, Strain P34048, NR-29436.
YCAT800	P78042	Clinical isolate, BSI	*C. albicans*	Clade 3	BEI Resources, NIAID, NIH: Candida albicans, Strain P78042, NR-29443.
YCAT801	P57055	Clinical isolate, BSI	*C. albicans*	Clade 3	BEI Resources, NIAID, NIH: Candida albicans, Strain P57055, NR-29439.
YCAT802	P57072	Clinical isolate, BSI	*C. albicans*	Clade 2	BEI Resources, NIAID, NIH: Candida albicans, Strain P57072, NR-29435.
YCAT803	P76055	Clinical isolate, BSI	*C. albicans*	Clade 2	BEI Resources, NIAID, NIH: Candida albicans, Strain P76055, NR-29441.
YCAT804	P76067	Clinical isolate, BSI	*C. albicans*	Clade 2	BEI Resources, NIAID, NIH: Candida albicans, Strain P76067, NR-29442.
YCAT805	P75010	Clinical isolate, BSI	*C. albicans*	Clade 11	BEI Resources, NIAID, NIH: Candida albicans, Strain P75010, NR-29437.
YCAT806	19F	Clinical isolate, VP, vaginal sample	*C. albicans*	Clade 1	BEI Resources, NIAID, NIH: Candida albicans, Strain 19F, NR-29449.
YCAT807	L26	Clinical isolate, VP, vaginal sample	*C. albicans*	Clade 1	BEI Resources, NIAID, NIH: Candida albicans, Strain L26, NR-29445.
YCAT808	P37039	Clinical isolate, VP, vaginal sample	*C. albicans*	Clade 1	BEI Resources, NIAID, NIH: Candida albicans, Strain P37039, NR-29451.
YCAT809	12C	Clinical isolate, VP, oral sample	*C. albicans*	Clade 1	BEI Resources, NIAID, NIH: Candida albicans, Strain 12C, NR-29444.
YCAT810	P37005	Clinical isolate, healthy individual, oral sample	*C. albicans*	Clade 1	BEI Resources, NIAID, NIH: Candida albicans, Strain P37005, NR-29447.

Table S2. Fungal strains used in the study.

Strain	Name	Description	Genotype	Species	Source
YCAT811	P37037	Clinical isolate, healthy individual, oral sample	*C. albicans*	Clade 1	BEI Resources, NIAID, NIH: Candida albicans, Strain P37037, NR-29450.
YCAT812	P78048	Clinical isolate, BSI	*C. albicans*	Clade 1	BEI Resources, NIAID, NIH: Candida albicans, Strain P78048, NR-29434.
YCAT1117	470140	Clinical Isolate, India	*C. auris*	Clade 1	Gift from Sarah Kidd; National Mycology Reference Center, Adelaide.
YCAT1193	B11203	Clinical Isolate, India	*C. auris*	Clade 1	CDC, Atlanta, USA.
YCAT1195	B11220	Clinical Isolate, Japan	*C. auris*	Clade 2	CDC, Atlanta, USA.
YCAT1196	B11221	Clinical Isolate, South Africa	*C. auris*	Clade 3	CDC, Atlanta, USA.
YCAT1198	B11244	Clinical Isolate, Venezuela	*C. auris*	Clade 4	CDC, Atlanta, USA.
YCAT607	CBS138	Type strain	*C. glabrata*	Clade 5	ATCC® 2001™
N/A	H99	Type strain	*C. neoformans*	VNI	Provided by Elizabeth Ballou
