# Supplementary material for: Gladiolin produced by pathogenic Burkholderia synergizes with amphotericin B through membrane lipid rearrangements
Source: mBio. 2024 Oct 18;15(11):e02611-24. doi: 10.1128/mbio.02611-24 (PMC11559049; doi:10.1128/mbio.02611-24)
Supplement: Legends — Supplemental legends. [file mbio.02611-24-s0003.pdf]

SUPPLEMENTAL DATA LEGENDS

Supplemental Results and Methods

Detailed information about the NMR procedure and results are available in the Supplementary data file.

Movie S1

AFM time course of POPC:ergosterol model lipids with ratios of 80%:20% under control conditions.

Movie S2

AFM time course of POPC:ergosterol model lipids with ratios of 80%:20% with 20 μg/ml gladiolin.

Movie S3

AFM time course of POPC:ergosterol model lipids with ratios of 80%:20% with 20 μg/ml AmpB.

Movie S4

AFM time course of POPC:ergosterol model lipids with ratios of 80%:20% with 10 μg/ml gladiolin and 10 μg/ml AmpB in combination.

Movie S5

AFM time course of POPC:ergosterol model lipids with ratios of 80%:20% with 20 μg/ml natamycin.

Movie S6

AFM time course of POPC:ergosterol model lipids with ratios of 80%:20% with 10 μg/ml gladiolin and 10 μg/ml natamycin in combination.

Table S1

FICIs of gladiolin/AmpB combination for the *C. albicans* clinical isolates tested in Figure S2.

Table S2

Fungal strains used in the study.

Dataset S1

Numerical data used to make the figures.
